# Integrated Analyses of Transcriptome and Chlorophyll Fluorescence Characteristics Reveal the Mechanism Underlying Saline–Alkali Stress Tolerance in *Kosteletzkya pentacarpos*

**DOI:** 10.3389/fpls.2022.865572

**Published:** 2022-05-06

**Authors:** Jian Zhou, Anguo Qi, Baoquan Wang, Xiaojing Zhang, Qidi Dong, Jinxiu Liu

**Affiliations:** ^1^School of Horticulture and Landscape Architecture, Henan Institute of Science and Technology, Xinxiang, China; ^2^Henan Province Engineering Center of Horticulture Plant Resource Utilization and Germplasm Enhancement, Xinxiang, China

**Keywords:** soil salinization, seashore mallow, photosynthetic function, sequencing, gene analysis

## Abstract

In recent years, soil salinization has become increasingly severe, and the ecological functions of saline–alkali soils have deteriorated because of the lack of plants. Therefore, understanding the tolerance mechanisms of saline–alkali-tolerant plants has become crucial to restore the ecological functions of saline–alkali soils. In this study, we evaluated the molecular mechanism underlying the tolerance of *Kosteletzkya pentacarpos* L. (seashore mallow) seedlings treated with 0.05 or 0.5% saline–alkali solution (NaCl: NaHCO_3_ = 4:1 mass ratio) for 1 and 7 days. We identified the key genes involved in tolerance to saline–alkali stress using orthogonal partial least squares regression analysis (OPLS-RA) based on both chlorophyll fluorescence indexes and stress-responsive genes using transcriptome analysis, and, finally, validated their expression using qRT-PCR. We observed minor changes in the maximum photochemical efficiency of the stressed seedlings, whose photosynthetic performance remained stable. Moreover, compared to the control, other indicators varied more evidently on day 7 of 0.5% saline–alkali treatment, but no variations were observed in other treatments. Transcriptome analysis revealed a total of 54,601 full-length sequences, with predominantly downregulated differentially expressed gene (DEG) expression. In the high concentration treatment, the expression of 89.11 and 88.38% of DEGs was downregulated on days 1 and 7, respectively. Furthermore, nine key genes, including *KpAGO4*, *KpLARP1C*, and *KpPUB33*, were involved in negative regulatory pathways, such as siRNA-mediated DNA methylation, inhibition of 5′-terminal oligopyrimidine mRNA translation, ubiquitin/proteasome degradation, and other pathways, including programmed cell death. Finally, quantitative analysis suggested that the expression of key genes was essentially downregulated. Thus, these genes can be used in plant molecular breeding in the future to generate efficient saline–alkali–tolerant plant germplasm resources to improve the ecological functions of saline–alkali landscapes.

## Introduction

Population growth and environmental degradation have caused soil salinization to become a global problem (Munns and Tester, 2008). Approximately 7% of the world’s land (over 900 million hectares) is threatened by salinization (Fang et al., 2021), among which northwest, north, and northeast China have significant distribution of saline–alkali soils. Unlike coastal saline soils, saline–alkali soils contain alkaline salts (such as NaHCO_3_), in addition to the neutral salt NaCl (Wang et al., 2008). Plants growing in saline–alkali soils are affected by factors, such as high pH, low water potential, high Na^+^ concentration, and drought, which cause biological toxicity (Alhdad et al., 2013) and severely hinder plant development.

Sowing saline–alkali-tolerant plants is a useful approach for improving the ecological functions of saline–alkali soils. Presently, plants with the potential of improving the quality of saline–alkali soils include *Puccinellia tenuiflora* (Guo et al., 2010), *Kochia scoparia* (Zhao, 2018), *Tamarix hispida* (Wang et al., 2014), and *Populus euphratica* (An et al., 2018).

*Kosteletzkya pentacarpos* L. (seashore mallow), formerly known as *Kosteletzkya virginica* (Liu et al., 2020, 2021), is a perennial halophyte belonging to the Malva genus of the Malvaceae family. It is naturally distributed on the salt marshy coasts of eastern United States, and is commercially used for the production of oil (Ruan et al., 2008), feed (Sun et al., 2019), medicines (Bai et al., 2015), and beauty products (Qin et al., 2015). The plant was introduced in China in 1993 as a candidate species for the development of coastal tidal flats (Xu et al., 1996). Previous studies on *K. pentacarpos* have focused on its saline–tolerance characteristics and mechanism (Blits and Gallagher, 1990a; Hasson and Poljakoff-Mayber, 1995; Guo et al., 2009b; Tang et al., 2015, 2020).

Several physiological adaptations add to the tolerance of *K. pentacarpos* to salt stress. Cations in *K. pentacarpos* are reverse transported across membranes, which establishes a favorable K^+^–Na^+^ relationship (Blits and Gallagher, 1990b,c). Its root system has a mechanism for Na^+^ repulsion and absorption (Blits and Gallagher, 1990c), endowing the plant with considerably high levels of salinity tolerance; Its hypocotyl callus can even grow in 240 mmol/L NaCl environments (Hasson and Poljakoff-Mayber, 1995). Under high-salinity stress, *K. pentacarpos* reduces biological toxicity by enhancing its ability to remove reactive oxygen species (Zhang et al., 2007).

In the early salinity stress stage, the expression of *K. pentacarpos* genes is upregulated and re-induced in the root system (Guo et al., 2009b). This involves ionic balance, plant growth and development, and signal transduction, which are mediated by peroxisome membrane proteins and ornithine transferase genes (Guo et al., 2009a). Wang et al. (2015a) cloned *KvP5CS1* from *K. pentacarpos* leaves, whose function in improving salinity tolerance by synthesizing proline to regulate cellular osmotic pressure was verified using a transgenic tobacco model (Wang H. Y. et al., 2019). Under 300 and 400 mmol/L NaCl conditions, proline concentrations in *K. pentacarpos* leaves were 9 and 27 times higher than that in the control, respectively, indicating that the regulation of osmotic pressure was closely related to its salinity tolerance (Wang et al., 2015b).

The heat shock protein gene *KvHSP70* is sensitive to NaCl stress and significantly improves the salinity tolerance of transgenic tobacco plants (Tang et al., 2020). Subsequently, the salinity stress-sensitive genes cloned from *K. pentacarpos*, such as the chloroplast small heat shock protein gene *KvHSP26* and the tonoplast intrinsic protein gene *KvTIP3*, are potential candidates for molecular plant breeding (Liu et al., 2020, 2021).

In 2011, *K. pentacarpos* was introduced in the saline–alkali beachhead soils of the Yellow River in northern China (Xu et al., 2013). However, there were major differences between the saline–alkali soils along the river and coastal saline soils. To date, studies on the saline tolerance of *K. pentacarpos* mainly focused on saline soils alone or salt-stressed environments. There have been no studies on the effects of mixed saline–alkali conditions and saline–alkali stress-mediating pathways, and the limited investigations have been restricted to the physiological level (Yan and Zhou, 2019; Zhou and Zhang, 2019; Dai and Zhou, 2020), which failed to fundamentally examine the tolerance mechanism of *K. pentacarpos* to mixed saline–alkali stress.

To address this issue, this study aimed to determine the key genes of *K. pentacarpos* that respond to saline–alkali stress using transcriptome sequencing, weighted gene co-expression network analysis (WGCNA), and orthogonal partial least squares regression analysis (OPLS-RA). The findings of this study will provide insights into the use of *K. pentacarpos* to improve saline–alkali soils and molecular plant breeding in the future.

## Materials and Methods

### Experimental Materials and Design

Seeds of *K. pentacarpos* were obtained from the Halophyte Research Laboratory of Nanjing University, which introduced *K. pentacarpos* from the Halophyte Biotechnology Center, University of Delaware, United States, in 1993.

Uniform and plump *K. pentacarpos* seeds were selected and soaked in concentrated sulfuric acid for 30 min, followed by rinsing with clean water and soaking for 24 h. Next, the seeds were placed on a wet towel and covered to induce germination. When one-third of the germinated seeds exhibited approximately 1 mm-long sprouts, they were sown in plastic cultivation bowls (diameter: 11 cm; height: 10 cm), with five seeds per bowl. Common garden soil (0.6 kg per bowl) was used for cultivation. A tray was arranged at the bottom of each bowl, and the bowls were placed in a greenhouse with day/night temperatures of 28/25°C. Then, 120 mL of water, based on specialized experimental determination, was added to each bowl per week. After all the seeds germinated, 120 mL of 25% Hoagland’s nutrient solution was added to provide nutrition once every 2 weeks. Furthermore, the water and the nutrient solution evenly permeated throughout the cultivation soil from the tray in this experiment.

According to the classification of China’s saline–alkali soil, the salt content of severe saline–alkali soil is 0.4–0.6% (Zhang, 2019). Therefore, in this study, salt concentration of the cultivation soil was set at 0.05 and 0.5%. Before the seedlings reached the age of 90 days, they were separately subjected to saline–alkali stress treatments for 1 and 7 days. Using the amount of cultivation soil in the bowls as the basis, NaCl and NaHCO_3_ were accurately weighed to a mass ratio of 4:1 to obtain total concentrations of 0.5 g/kg (0.05%) and 5 g/kg (0.5%). The saline–alkali mixture was dissolved in 120 mL of distilled water, placed in the tray at the base of each bowl, and allowed to permeate evenly throughout the cultivation soil. All seedlings were sampled and measured at 90 days of age. In this experiment, seedlings cultivated using ordinary garden soil served as the control (CK). The treatment groups were as follows: (i) Tr1: 0.05% saline–alkali solution for 1 day; (ii) Tr2: 0.05% saline–alkali solution for 7 days; (iii) Tr3: 0.5% saline–alkali solution for 1 day; and (iv) Tr4: 0.5% saline–alkali solution for 7 days. Each treatment group consisted of six cultivation bowls.

### Measurement of Chlorophyll Fluorescence Characteristics

The chlorophyll fluorescence parameters were measured using a YAXIN 1161G chlorophyll fluorometer (Beijing Yaxinliyi Science and Technology Co., Ltd., Beijing, China). Intact leaves from the middle–upper section of the seedlings were selected and darkened for 30 min using clamping blade clips before testing. The leaves were treated with saturated pulsed light at 3,000 μmol⋅m^–2^⋅s^–1^ for 1 s followed by actinic light at 1,000 μmol⋅m^–2^⋅s^–1^ for 9 s. The light-induced curve was then used to measure the initial fluorescence (F_0_) and other indicators of chlorophyll fluorescence. From each treatment group, three cultivation bowls were randomly selected, and each bowl was tested five times to obtain the average value. Indicators were measured thrice.

### RNA Extraction and Analysis

Leaves from the middle–upper section of the seedlings and some tender stems were collected and immediately frozen using liquid nitrogen at −80°C for storage. From each treatment group, three cultivation bowls were selected for analyses. After extracting total RNA using a Takara RNA Preparation Kit (Takara Bio, Dalian, China), RNA concentration and quality were determined using a Nanodrop ND-1000 spectrophotometer (NanoDrop Technologies, DE, United States) and Agilent 2100 Bioanalyzer system (Agilent Technologies, CA, United States), respectively.

### Full-Length Transcriptome Sequencing and Data Analysis

Full-length (FL) cDNAs were synthesized using a SMARTer™ PCR cDNA Synthesis Kit (Takara Bio, Dalian, China), and cDNA length (1–6 kb) was determined and screened using a BluePippin™ Size-Selection System (Sage Science, Beverly, MA, United States). Next, a DNA Template Prep Kit 2.0 (Pacific Biosciences, Menlo Park, California, United States) was used to establish the SMRTbell library before performing single-molecule real-time (SMRT) sequencing on the PacBio RSII platform (Pacific Biosciences, Menlo Park, California, United States).

The polymerase reads that the length is less than 50 bp, and the accuracy is less than 0.90, were filtered according to the standard procedures of the SMRT Analysis Software package, and sub-sequences shorter than 50 bp were removed to obtain insert reads. The Iso-Seq module of the SMRT Link software was used to iteratively cluster similar full-length (FL) non-chimeric (FLNC) sequences. Consensus isoforms were obtained and further corrected to obtain high-quality transcriptomes with accuracies above 99%. Subsequently, the corresponding Illumina RNA-seq data were input in the Proovread 2.13.841 software to correct for low-quality consensus sequences, thereby increasing sequence accuracy. Finally, the CD-HIT 4.6.142 software was used to eliminate redundant sequences (Li and Godzik, 2006), resulting in a high-quality transcriptome database.

### Second-Generation Transcriptome Sequencing and Data Analysis

The operating instructions of the NEBNext^®^ Ultra™ RNA Library Preparation Kit (NEB, Beverly, MA, United States) were followed to generate a second-generation sequencing cDNA library. After purification of the cDNA fragments using the AMPure XP system, the Agilent 2100 Bioanalyzer was used to evaluate the quality of the library. After the quality was ascertained, cDNA library sequencing was performed on the Illumina HiSeq 2500 platform (Illumina, San Diego, CA, United States) to derive paired-end reads.

The raw data were processed to eliminate the sequencing adapters and primer sequences to obtain clean reads before the value of fragments per kilobase of exon per million fragments mapped (FPKM) was used to measure the level of gene expression. The DESeq R software package of the Bioconductor platform was then run to analyze the differential expression between the transcriptomes of the various treatment groups (Anders and Huber, 2010). Differentially expressed genes (DEGs) were screened using fold change ≥2 and false discovery rate (FDR) <0.01 as the standards.

The identified DEGs were clustered using k-means method, and then used for KEGG enrichment analysis. The KOBAS software was used to test the statistical enrichment of DEGs in KEGG pathways (Mao et al., 2005). The hypergeometric test was used to analyze pathway enrichment based on the KEGG pathway database as the unit. The results were compared with the transcriptome background to identify enriched pathways from the differentially expressed transcriptomes.

Using the NCBI database,^1^ a homology search and comparison (*E*-value ≤ 1e-5) of the key genes (FL sequences) selected from the DEGs was performed. Based on query coverage, identity percentage, and E-value of matched nucleobases, the comparison result ranked first in the database were then screened.

### Weighted Gene Co-expression Network Analysis of Differential Genes

The WGCNA R software package (Langfelder and Horvath, 2008) was used to construct a weighted gene co-expression network. The WGCNA analysis was performed on the DEGs with FPKM values ≥1 and coefficient of variation between treatments ≥0.5 for a total of 15 transcriptome samples (5 treatments, each with 3 replicates). After threshold screening and determination of the weighting coefficient β, the original scaled relationship matrix was subjected to power processing to obtain an unscaled adjacency matrix. Considering the correlation of expression patterns between a gene and other genes in WGCNA analysis, the adjacency matrix was further transformed into a topological overlap matrix (TOM). Based on topological dissimilarity matrix (diss TOM = 1-TOM), dynamic shearing algorithm was used for gene clustering and module division. Furthermore, the minimum number of genes in a module was 30 (min Module Size = 30), the threshold for merging similar modules was 0.1327 (minimum Height for Merging Modules = 0.1327), and the network type was “Unsigned” in this analysis.

The genes were selected as module members according to the kME value > 0.7. Some modules, which exhibited high correlations with sample traits, were selected from the heatmap, and their gene co-expression visualization network diagrams were constructed using the Cytoscape 3.7.2 software.

### Quantitative Expression of Real-Time Fluorescence in Selected Genes

Leaves from the middle–upper section and tender stems were mixed following the aforementioned experimental design. Next, a SteadyPure Plant RNA Extraction Kit (Hunan Accurate Bio-Medical Co., Ltd., Changsha, China) was used to extract RNA for quality inspection according to the manufacturer’s instructions. After quality testing, a PrimeScript™ RT reagent kit with gDNA Eraser (Perfect Real Time) (Takara Bio, Dalian, China) was used to synthesize cDNA by reverse transcription.

A CFX96 real-time fluorescence quantitative PCR system (Bio-Rad Laboratories, Inc., California, United States) was used for qRT-PCR analysis. The reagent test kit used was the TB Green^®^ Premix EX Taq™ II (Tli RNase H Plus) (Takara Bio, Dalian, China), the dye was TB Green, and the internal reference gene was β-actin. The primer designing tool of NCBI was used to design the fluorescence quantitative PCR primers. Relative gene expression was analyzed using the 2^–ΔΔ*CT*^ method (Livak and Schmittgen, 2001) with three replicates.

### Statistics

SPSS 21.0 was used to perform Duncan’s multiple range test at a significance level (α) of 0.05; SIMCA 14.1 was used to perform OPLS-RA.

## Results

### Fluorescence Characteristics of *Kosteletzkya pentacarpos* Seedlings Under Saline–Alkali Stress

**FIGURE 1:**
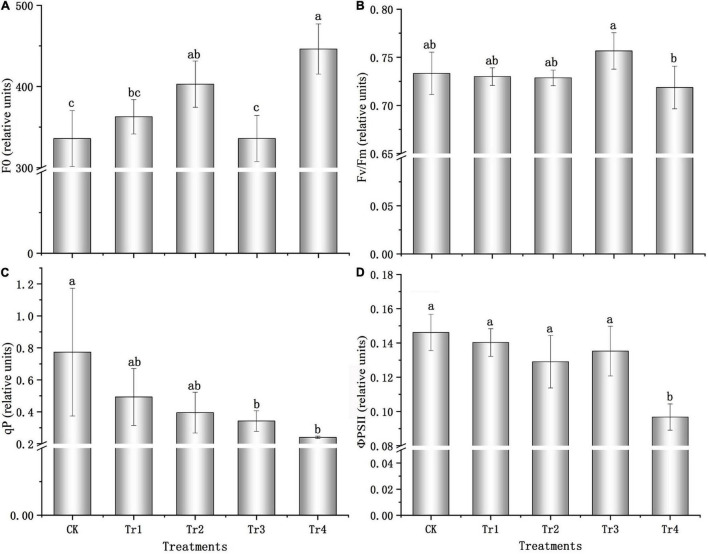
Chlorophyll fluorescence characteristics of *K. pentacarpos* seedlings under saline–alkali stress. **(A)** Initial fluorescence (F_0_). **(B)** Maximum photochemical efficiency (F_*v*_/F_*m*_). **(C)** Photochemical quenching coefficient (qP). **(D)** PSII quantum yield (ΦPSII). Vertical bars in the figure indicate mean ± SD (*n* = 3). Different letters indicate significant differences at *P* < 0.05.

The F_0_ of seedlings increased with prolonged treatment with 0.05 and 0.5% saline–alkali solutions. All treatments exhibited F_0_ values greater than that of the control, and the F_0_ value was 32.85% higher than that of the control, with a significant difference under the high-concentration condition (*P* = 0.001, see Figure 1A) on day 7. Compared to the control, the maximum photochemical efficiency (F_*v*_/F_*m*_) was relatively stable and changed slightly under saline–alkali conditions (see Figure 1B). However, F_*v*_/F_*m*_ significantly decreased under prolonged high-concentration condition (*P* = 0.022), and the value on day 7 was 5.02% lower than that on day 1. The photochemical quenching coefficient (qP) and PSII quantum yield (ΦPSII) also presented similar patterns (see Figures 1C,D): under the 0.05 and 0.5% saline–alkali conditions, both parameters decreased with prolonged treatment. The variations in qP and ΦPSII were significant under the 0.5% saline–alkali condition after 7 days (*P* = 0.010, *P* = 0.000), and qP and ΦPSII values decreased by 68.94 and 33.80%, respectively, compared with the respective control groups.

### Analysis of *Kosteletzkya pentacarpos* Transcriptome Characteristics Under Saline–Alkali Stress

The SMRT sequencing technique was used to determine the FL transcriptomes of *K. pentacarpos* seedlings. An SMRT cell was used to establish an FL cDNA library with a sequence length of 1–6 kb (Table 1). Subreads smaller than 50 bp in length were filtered, yielding 18.95 G of clean data. A total of 410,351 circular consensus sequences (CCS) were extracted based on the criteria of full passes ≥3 and sequencing accuracy >0.9, with sequence length distributed between 1 and 3 kb (Supplementary Figure 1A). After removing the cDNA primer and polyA sequences from the CCS, 383,234 FLNC sequences were obtained, most of which were 1–3 kb in length (Supplementary Figure 1B). Following iterative clustering, 96,419 consensus isoforms were obtained, with the majority of the transcriptomes being approximately 2-kb long (Supplementary Figure 1C). Further correction yielded 93,218 high-quality consensus isoforms, the accuracies of which were above 99%. Finally, highly similar sequences were merged, and redundancies were removed, leaving 54,601 non-redundant sequences.

**TABLE 1:** PacBio iso-seq output statistics for *K. pentacarpos* seedlings.

CCS data					
Samples	cDNA size	CCS number	Read bases of CCS	Mean read length of CCS	Mean number of passes
F01	1–6K	410351	831995069	2027	24

**FLNC data**					
Samples	Number of CCS	Number of undesired primer reads	Number of filtered short reads	Number of FLNC reads	FLNC%
F01	410351	19467	33	383234	93.39%

**Clustering and redundance removal**					
Samples	Number of consensus isoforms	Average consensus isoforms read length	Number of polished HQ isoforms	Percent of polished HQ isoforms (%)	Non-redundant consensus isoforms
F01	96419	2109	93218	96.68%	54601

**FIGURE 2:**
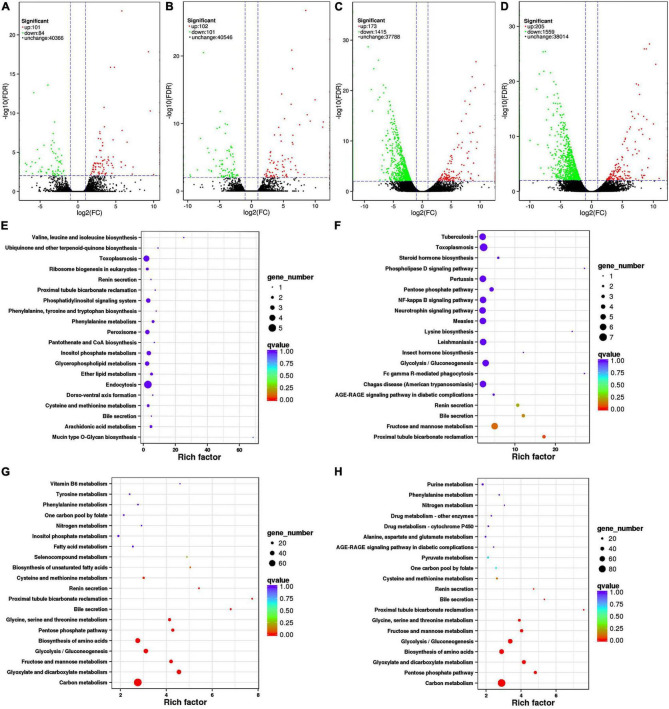
Volcano plots and pathway enrichment of differently expressed transcripts in *K. pentacarpos* seedlings under saline–alkali stress. **(A–D)** Volcano plots of CK-Tr1, CK-Tr2, CK-Tr3, and CK-Tr4. Green, red, and black dots represent down- and upregulated differential and non-differential expression, respectively. **(E–H)** Pathway enrichment of CK-Tr1, CK-Tr2, CK-Tr3, and CK-Tr4. The larger the enrichment factor, the more significant the enrichment level; the smaller the *q*-value, the more reliable the enrichment significance; and the larger the dot, the greater the number of transcriptomes.

In this experiment, differential expression in the transcriptomes of *K. pentacarpos* seedlings was not evident following 0.05% saline–alkali treatment (Figures 2A,B). The number of DEGs on day 1 and 7 were 185 and 203, respectively. Under 0.5% saline–alkali treatment, differential expression in their transcriptomes became evident, with 1,588 and 1,764 DEGs on days 1 and 7, respectively. Among these, downregulated DEGs were predominant (Figures 2C,D) and accounted for 89.11 and 88.38% of the total expression on days 1 and 7 of 0.5% saline–alkali treatment, respectively. These results revealed that saline–alkali concentrations considerably affected *K. pentacarpos* seedlings than treatment duration.

The top 20 pathways with the smallest q values are shown in Figures 2E–H for the four treatments. Under 0.05% saline–alkali treatment, the enrichment factors of each pathway were small, but the q value was larger on day 1. The pathways were mainly enriched in the biosynthesis and endocytosis of ubiquinone and terpenoid-ubiquinone (Figure 2E). When the seedlings were subjected to stress for 7 days, a small portion of the pathway enrichment factors increased, while the q value became smaller. Most pathways were similar to those on day 1 and were mainly enriched in pathways, such as phagocytosis and metabolism of fructose and mannose (Figure 2F). The pathway enrichment conditions on days 1 and 7 were similar with 0.5% saline–alkali treatment. The enrichment factors of the various pathways increased significantly compared with that of low-concentration treatment, but the q value was small. The number of enriched transcriptomes also increased significantly. Enrichment occurred in various pathways, including those of carbon metabolism, amino acid biosynthesis, and fructose and mannose metabolism (Figures 2G,H).

### Weighted Gene Co-expression Network Analysis of Differential Genes in *Kosteletzkya pentacarpos* Under Saline–Alkali Stress

**FIGURE 3:**
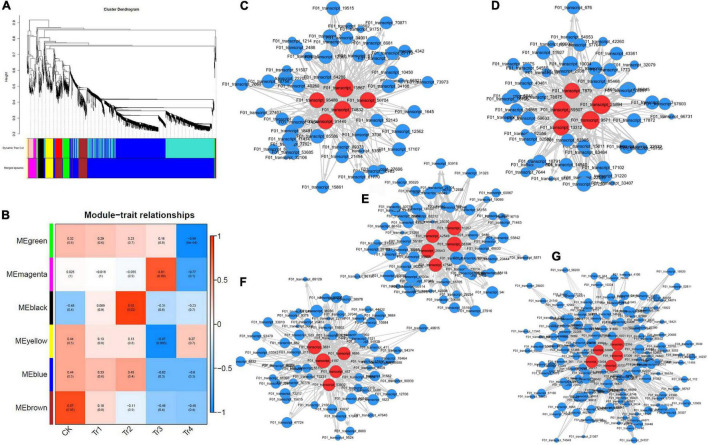
WGCNA characteristics of differently expressed transcripts in *K. virginica* seedlings under saline–alkali stress. **(A)** Clustering dendrograms of genes and detected modules. **(B)** Heatmap of the correlation between modules and traits. **(C–G)** Gene co-expression networks of Meblack, Mebrown, Megreen, Memagenta, and Meyellow. Red dots represent core genes.

We used kME values to evaluate the existence of effective connectivity between key genes and identify module members. In this experiment, DEGs with kME >0.7 were selected as module members, and similar modules were merged after their eigenvectors were calculated, resulting in six gene co-expression modules (Figure 3A). The modules had 52 (Memagenta) to 1,370 (Meblue) DEGs. The expression patterns of DEGs in the same module were similar and downregulated.

The modules–traits correlation heatmap (Figure 3B) reflected the correlation between genes in samples with related traits and the modules to which they belonged. The greater the absolute value, the stronger the correlation. Red and blue colors indicate positive and negative correlations, respectively. In this experiment, five gene modules were highly correlated with the saline–alkali stress in *K. pentacarpos*, with all their correlation coefficients being > 0.80. Among them, Memagenta (*r* = 0.81), Mebrown (*r* = 0.87), and Meblack (*r* = 0.92) were positively correlated with CK, Tr2, and Tr3, respectively; Meyellow (*r* = −0.97) and Megreen (*r* = −0.99) were negatively correlated with Tr3 and Tr4, respectively. The WGCNA visualization diagrams for the five modules were generated (Figures 3C–G), and the top five genes with the highest kME values in each module were selected as key genes for that module (marked in red, see Supplementary Table 1).

### Screening of Key Genes in *Kosteletzkya pentacarpos* Seedlings That Responded to Saline–Alkali Stress

**FIGURE 4:**
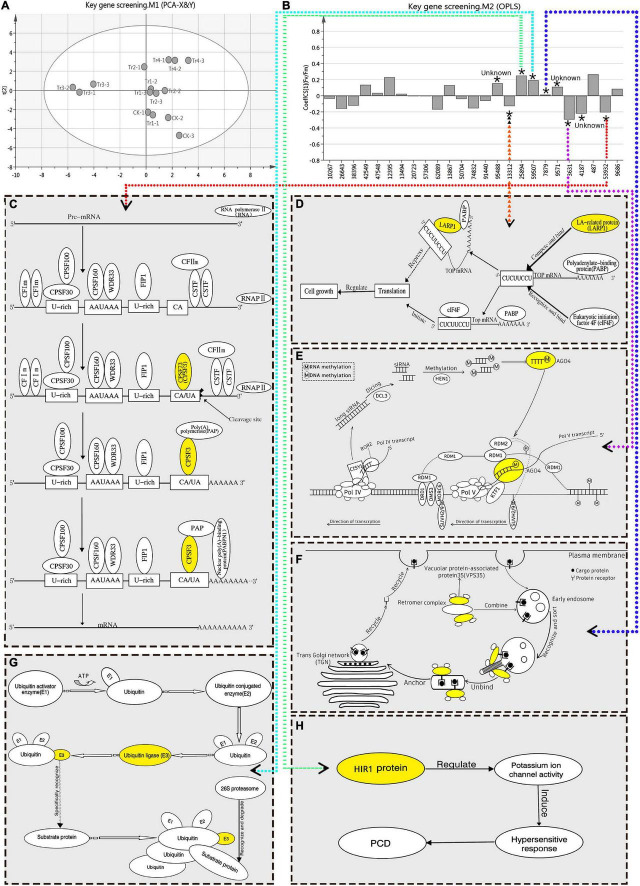
OPLS-RA conduction and filtration of key genes responsive to saline–alkali stress in *K. pentacarpos* seedlings. **(A)** Sample score chart of PCA. **(B)** OPLS-RA model diagram. In this model, “*” indicates that the VIP value of the corresponding transcript is >1 in this model, “Unknown” indicates that function of the corresponding gene is not clear, the numbers on the x-axis represent transcript ID of 25 core genes, and dotted arrows in different colors point to functional maps of the corresponding genes. **(C–H)** Function diagrams of the key genes filtered using the OPLS-RA model, including *KpCPSF3* (**C**, diagram **C** refers to this literature; Xu et al., 2021), *KpLARP1C* (**D**, diagram **D** refers to this literature; Philippe et al., 2018), *KpAGO4* (**E**, diagram **E** refers to the literatures; Pikaard et al., 2012; Matzke and Mosher, 2014), *KpVPS35A* (**F**, diagram **F** refers to this literature; Song et al., 2016), *KpPUB33* **(G)**, *KpHIR1* **(H)**.

F_*v*_/F_*m*_ reflects the potential maximum light conversion efficiency of plants, and can indicate their overall health status (Bjorkman and Demming, 1987). Therefore, it is an important indicator of the impact of environmental stress on photosynthetic performance. In this study, OPLS-RA was performed on the F_*v*_/F_*m*_ (Y) of *K. pentacarpos* and the FPKM value (X) of the selected 25 key genes. The degree of influence of each factor over photosynthetic performance was analyzed using the VIP value, which was the basis for screening the key genes. After fitting the principal component analysis model (*R*^2^X = 0.504, Q^2^ = 0.149), the score chart of the samples (Figure 4A) revealed that the 15 sample groups were normally distributed with no abnormalities. The regression model was established using OPLS-RA fitting (*R*^2^X = 0.625, *R*^2^Y = 0.921, *Q*^2^ = 0.542).

The VIP value of the model indicated the degree of influence that the relevant factors exhibited on Y. The selection criterion, based on the requirements stipulated in the SIMCA user guide, was that the VIP value must be > 1. After evaluation, nine DEGs in the *K. pentacarpos* seedlings were found to have VIP values > 1 (Supplementary Table 2) and were selected as key genes that responded to saline–alkali treatments (Figure 4B).

The FL cDNA sequences (Supplementary Table 3) were used to perform homology comparisons with the NCBI database. Among them, the functions of three genes was unknown, while those of the remaining six were known. The IDs of their transcriptome sequence were F01_transcript_53932, F01_transcript_13312, F01_transcript_3631, F01_transcript_ 7879, F01_transcript_59507, and F01_transcript_25894. After comparison, these six genes were highly homologous to plants, such as *Hibiscus syriacus* and *Gossypium hirsutum*, both of which belong to the Malvaceae family. These genes were predicted to be *KpCPSF3*, *KpLARP1C*, *KpAGO4*, *KpVPS35A*, *KpPUB33*, and *KpHIR1* (Figures 4C–H). The specific comparisons are given in Table 2.

**TABLE 2:** Sequence match in NCBI database and functional analysis of key differentially expressed genes in *K. pentacarpos* seedlings under saline–alkali stress.

Transcript ID	Gene type	Functional description	Matching species	Query coverage (%)	Identity percentage (%)	*E-*value	Accession
F01_transcript_ 53932	*KpCPSF3*	The encoded protein binds to pre-mRNA, performs precise cleavage, and assists in the polymerization of poly(A) to complete the processing of mature mRNA.	*Hibiscus syriacus*	78.00	92.48	0.00	XM_039209821.1
F01_transcript_ 13312	*KpLARP1C*	The encoded protein competes with eukaryotic initiation factor 4F to bind to 5′ terminal oligopyrimidine mRNA (TOP mRNA), inhibit its translation, and then regulate cell growth.	*Gossypium hirsutum*	40.00	86.57	0.00	XM_016852313.2
F01_transcript_ 3631	*KpAGO4*	AGO4 protein binding to siRNA (short interfering RNA) mediates histone methylation and non-CG site DNA methylation in chromatin	*H. syriacus*	92.00	91.95	0.00	XM_039136791.1
F01_transcript_ 7879	*KpVPS35A*	This gene is mainly involved in endocytosis, where VPS35 binds to cargo proteins and transports them to the trans -Golgi network region.	*H. syriacus*	91.00	94.18	0.00	XM_039152238.1
F01_transcript_ 59507	*KpPUB33*	After binding to ubiquitin, U-box protein can specifically recognize and bind to substrate proteins, and these proteins are marked by ubiquitin chains and then degraded by the 26S proteome.	*H. syriacus*	83.00	91.81	0.00	XM_039139593.1
F01_transcript_ 25894	*KpHIR1*	The protein encoded by this gene can induce hypersensitivity response to external stress by regulating activity of potassium channels, and thus initiates programmed cell death.	*H. syriacus*	92.00	89.44	0.00	XM_039207218.1

Functional analysis revealed that the key genes were involved in regulating pathways, such as vesicular transport (*KpVPS35A*), programmed cell death (PCD; *KpHIR1*) induction, transcription levels (*KpCPSF3* and *KpAGO4*), translation levels (*KpLARP1C*), and post-translational protein levels (*KpPUB33*) (see Table 2). Most genes exhibited negative regulatory effects.

### qRT-PCR Analysis of Key Genes of *Kosteletzkya pentacarpos*

**FIGURE 5:**
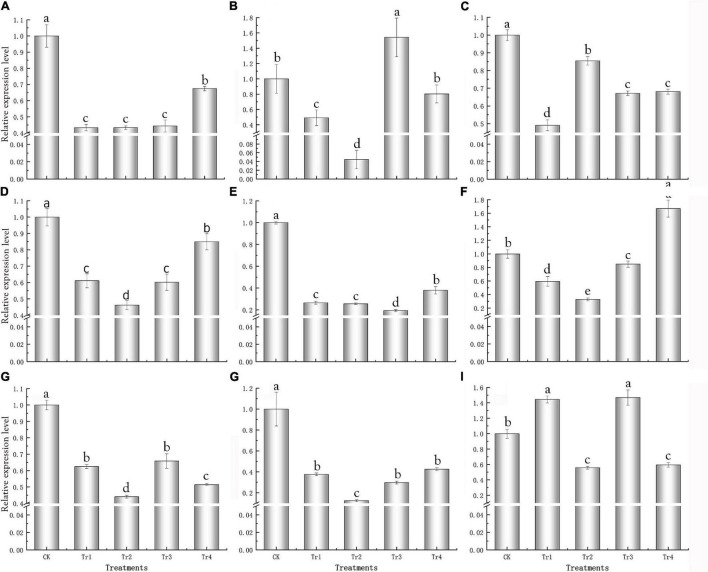
qRT-PCR analysis of key genes responsive to saline–alkali stress in *K. pentacarpos* seedlings. **(A)** F01_transcript_53932 (*KpCPSF3*), **(B)** F01_transcript_13312 (*KpLARP1C*), **(C)** F01_transcript_3631 (*KpAGO4*), **(D)** F01_transcript_7879 (*KpVPS35A*), **(E)** F01_transcript_59507 (*KpPUB33*), **(F)** F01_transcript_25894 (*KpHIR1*), and **(G–I)** F01_transcript_95488, F01_transcript_9571, and F01_transcript_4187 (their function is unknown).

Specific primers were designed according to the FL transcriptome sequences (Supplementary Table 4) for qRT-PCR analysis of the nine key genes. For most treatments, the expression levels of the key genes were significantly lower than those of the control and were downregulated (Figure 5); this was consistent with the transcriptome results.

Among the nine genes, the expression patterns of five genes—F01_transcript_53932, F01_transcript_7879, F01_transcript_59507, F01_transcript_25894, and F01_transcript _9571—were similar. Compared to the control, gene expression gradually decreased under Tr1 and Tr2 (low saline–alkali treatments). Nonetheless, gene expression initially decreased but recovered under Tr3 and Tr4 (high saline–alkali treatments), despite being lower than that of the control (Figures 5A,D–H). However, their expression levels under Tr2 was the lowest among all treatments, and significantly decreased by 56.65, 53.80, 67.16, and 87.51% compared with those of their corresponding controls (*P* = 0.000).

The expression patterns of F01_transcript_13312, F01_transcript_95488, and F01_transcript_4187 were similar; under prolonged saline–alkali treatments, the expression levels of these three genes decreased. The expression levels of these genes inf most treatment groups were lower than those in the control, and only few genes exhibited expression levels greater than the control for the treatment groups on day 1 (Figures 5B,G,I), which under the Tr2 treatment were the lowest and 95.54, 55.92, and 44.14% lower than those of their respective controls (*P* = 0.000). This anomaly might be caused by an emergency response to saline–alkali stress. Under Tr1 and Tr2, the expression of F01_transcript_3631 increased with time, and the value under Tr1 significantly decreased by 50.90% compared with that of the control (*P* = 0.000), whereas under Tr3 and Tr4, its expression levels were relatively stable but consistently lower than that of the control (Figure 5C).

## Discussion

### Characteristics of the Photosynthetic Functions of *Kosteletzkya pentacarpos* Seedlings Under Saline–Alkali Stress

In this study, the Fv/Fm of seashore mallow was stable under saline–alkali stress, and the Fv/Fm value of each treatment was not significantly different from that of control plants. However, F0, qP, and ΦPSII changed significantly in the later stages of high-concentration saline–alkali treatment compared with their respective controls, and the variations were relatively small in other treatments.

The decrease in Fv/Fm of the stressed seedlings can be attributed to the inactivation of the PSII reaction center (Da̧browski et al., 2015) or blockage of the photosynthetic electron transport chain (Tuba et al., 2010). However, the difference between the F_*v*_/F_*m*_ values of the treated plants and the control was not significant under Tr4, indicating that the photosynthetic performance of the *K. pentacarpos* seedlings was relatively stable under saline–alkali stress conditions. However, qP was used to reflect the photosystem pressure due to the excess excitation energy of PSII (Öquist and Huner, 1993). With increasing saline–alkali concentrations, the qP of the *K. pentacarpos* seedlings decreased with time, indicating that the pressure of excitation energy gradually increased on photosystem and the photosynthetic function was affected (Öquist and Huner, 1993). As for the electron transport chain, the ΦPSII reflected the working status of PSII (Li and Feng, 2004). In this study, the variation was similar to that of F_*v*_/F_*m*_, indicating that the PSII electron transport chain was relatively normal in the early stage, but electron transfer was blocked to weaken photosynthetic function in the later stage. Based on chlorophyll fluorescence characteristics, photosynthetic performance of the seedlings was relatively stable, and *K. pentacarpos* showed strong tolerance to saline–alkali stress.

### Impact of Negative Regulation on *Kosteletzkya pentacarpos* Response to Saline–Alkali Stress

Plants must finely regulate their gene expression in response to environmental stress. Although previous studies have focused on positive regulatory mechanisms (Cao et al., 2017; Pang et al., 2017; Lu et al., 2019), recent studies have paid increasing attention to negative regulation. In this study, downregulated DEGs accounted for 89.11 and 88.38% of the expression under Tr3 and Tr4, respectively, with negative regulation being predominant. Three negative regulatory pathways, involving the key genes of *K. pentacarpos*, were involved in responding to saline–alkali stress: (i) LARP1 inhibited the translation of 5′-terminal oligopyrimidine mRNAs (TOP mRNAs) (Philippe et al., 2018); (ii) AGO4 -mediated DNA methylation through siRNA interaction (Pikaard et al., 2012; Matzke and Mosher, 2014); and (iii) the plant U-box33 recognized and labeled target proteins for degradation by the 26S proteasome in the ubiquitin pathway (Jin et al., 2007).

The 5′-TOP mRNAs, a class of eukaryotic mRNA family, contains proteins that regulate cell growth (Philippe et al., 2018), whose translation is regulated by the eukaryotic promoter 4F (eiF4F). Its translational abilities can be inhibited by LARP1, which competes to bind with TOP mRNAs (Tcherkezian et al., 2014). Fonseca et al. (2015) used RNA interference techniques to reduce the levels of LARP1, thereby alleviating its inhibitory effects on TOP mRNA translation. However, target of rapamycin (TOR) specifically controls the translation of 5′-TOP mRNAs by the putative TOR substrate, LARP1. Furthermore, the regulatory pathway of TOR–LARP1–5′-TOP is conserved in plants (Scarpin et al., 2020). In this study, *KpLARP1C* expression decreased with prolonged saline–alkali treatment, and its expression in most treatment groups was lower than that of the control. It is speculated that the decreased expression of *KpLARP1C* may reduce competition and the inhibition of TOP mRNA translation and promote cell growth, thereby enhancing the tolerance of *K. pentacarpos* seedlings to saline–alkali stress.

AGO4 has been mainly reported in studies of plant resistance to diseases (Brosseau et al., 2016). AGO4 achieves transcriptional silencing of genes through DNA methylation (Raja et al., 2008; Duan et al., 2015), leading to the regulation of plant responses to biotic and abiotic stress (Pu et al., 2021). *Arabidopsis thaliana* mutant, which over-expresses *AtAGO4*, is more likely to be infected by *Pseudomonas syringae* (Agorio and Vera, 2007), while the double mutant of *AtAGO4* and *AtAGO2* is susceptible to the tobacco rattle virus (Ma et al., 2015). AGO4 induces nucleic chromatin modifications and prevents recessive transcription to maintain or activate the expression of stress-responsive genes (Al et al., 2017), which regulate physiological pathways, such as jasmonic acid signaling pathway (Prashanm et al., 2020). As for hypoxia, AGO1 in *Arabidopsis* coordinates AGO4, which represses the expression of HR4 by DNA methylation to regulate stress tolerance (Loreti et al., 2020). Under saline–alkali stress, the expression of *KpAGO4* was lower than that of the control plants., indicating that the decreased expression of *KpAGO4* may weaken the inhibition of DNA methylation and transcriptional gene silencing. Then, the function of related genes mediated by *KpAGO4* could be activated to respond to stress (Al et al., 2017), thereby improving the tolerance of *K. pentacarpos* seedlings to saline–alkali stress. This, in turn, maintained the stability of their photosynthetic function.

The ubiquitin system can selectively degrade proteins related to stress response, growth, and development of plants to adapt to environmental stress (Varshavsky, 1997). The plant U-box (PUB) protein is a type of ubiquitin-linked enzyme, E3, that specifically identifies target proteins (Zhou and Zeng, 2017), enabling plants to respond to stress. Sixty-six *StPUB* genes have been identified in potato, and 200 proteins are modified, including 25 differential ubiquitination modification sites under PEG-induced drought (Tang et al., 2022). *Arabidopsis thaliana* proteins, PUB22 and PUB23, act on RPN12a and cooperate to negatively regulate drought-stress responses through the drought signaling pathway (Cho et al., 2008; Seo et al., 2012). Similarly, *AtPUB11* is a negative regulator of drought tolerance, which degrades LRR1 and KIN7 (Chen et al., 2021). *Capsicum frutescens CaPUB1* gene, which was heterologously transferred into rice, negatively regulated rice response to drought-stress and decreased drought-tolerance of rice (Min et al., 2016). Under salinity stress, *A. thaliana* protein PUB30 degraded BKI1 through ubiquitination and negatively regulated the salinity tolerance of plants (Zhang et al., 2017). After the *Pohlia nutans PnSAG1* gene was heterologously overexpressed in *A. thaliana*, the sensitivity of transformed plants to salinity stress increased, indicating negative regulation (Wang J. et al., 2019). In this study, *KpPUB33* expression was significantly downregulated in stressed *K. pentacarpos* plants. This indicates that a decrease in *KpPUB33* expression maybe alleviate the ubiquitin-mediated degradation of target proteins, and then maintain the normal functions of the target proteins, thereby improving saline–alkali tolerance of *K. pentacarpos*.

### Significance of Programmed Cell Death in *Kosteletzkya pentacarpos* Response to Saline–Alkali Stress

Plant PCD can be classified as apoptotic or autophagic (Huang and Fu, 2010). Apoptotic PCD often occurs in stress-induced hypersensitivity reaction (HR), such as heavy metal or salinity stress (Pan et al., 2001; Liu et al., 2007). Hypersensitivity-induced response (HIR) genes can induce HR responses and participate in the regulation of ion channels and cell death (Zhou et al., 2010). Overexpression of the *C. frutescens CaHIR1* in *A. thaliana* led to tissue necrosis similar to HR and improved plant resistance to bacterial and fungal infections (Jung and Hwang, 2007). The expression of *Arachis hypogaea AhHIR* was significantly decreased under low-temperature stress, which increased with time (Liu et al., 2014). This observation was similar to that of *K. pentacarpos KpHIR1* under saline–alkali stress. The expression of *KpHIR1* decreased under Tr3 but increased to 66.91% compared to the control value under Tr4 (*P* = 0.000), whereas its expression continuously decreased under Tr1 and Tr2. Downregulation of the expression of HIR gene was conducive to reducing cell mortality (Liu et al., 2014), whereas the upregulation of its expression promoted apoptosis-like PCD to form a barrier of dead cells (Liu et al., 2007), which prevented further tissue damage by the salt ions (Liu et al., 2007). This is the potential mechanism by which *K. pentacarpos* seedlings increase tolerance to saline–alkali stress.

Autophagic PCD is induced by stress, such as drought, salinity, and nutrient deficiency, where the endoplasmic reticulum is involved in regulating and inducing cell death (Huang and Fu, 2010). During PCD, endoplasmic reticulum recycles nutrients of damaged cells to supply them to other cells for survival. Phagocytes, however, reuse these nutrients through autophagy and vesicular transport (Song et al., 2016). The VPS35 protein in the vesicular transport complex Retromer specifically identifies the cargo protein, transports it to the vesicles of the Golgi reverse membranes, and then packages and exports it (Song et al., 2016), thereby ensuring reuse of the protein. Therefore, the Retromer complex could regulate the identification of dead cells by phagocytes through the cargo protein CED-1, and to recycle more nutrients (Yamanaka and Ohno, 2008). Under high-concentration saline–alkali stress, *KpVPS35A* expression increased with time, indicating that the ability to identify and transport the cargo protein was improved by VPS35. This led to improved precise identification of the PCD cells, which facilitated the recycle and reuse of their nutrients and maintained the vitality of other cells to help *K. pentacarpos* seedlings survive saline–alkali conditions.

## Conclusion

Based on the results in this study, we conclude that under saline–alkali stress, the photosynthetic performance of seashore mallow was relatively stable, the seedlings exhibited strong tolerance, and the saline–alkali concentration was more influential than the duration of exposure. The expression of the DEGs was mainly downregulated, indicating that *K. pentacarpos* responded to saline–alkali stress through a negative regulatory pathway. Nine key genes in saline–alkali-stressed *K. pentacarpos* seedlings were screened using WGCNA and OPLS-RA, six of which had known functions and were mainly involved in negative regulatory pathways, such as ubiquitin degradation, siRNA-mediated DNA methylation, and inhibition of TOP mRNAs translation, and other pathways, including vesicle transport and PCD. Using qRT-PCR analysis, the expression of the nine key genes showed a declining trend, which was consistent with the transcriptomic data.

The key genes screened in this study need further functional studies in model plants. Besides functional tests, both degraded target proteins and methylated target genes require further investigations to determine their roles in regulatory pathways. Additionally, the key genes can also be used for plant molecular breeding to generate more saline–alkali–tolerant plant germplasm resources in the future. This will help restore saline-alkali lands to improve their ecological functions and alleviate the development of soil salinization in China and other countries.

## Data Availability Statement

The datasets presented in this study can be found in online repositories. The names of the repository/repositories and accession number(s) can be found below: https://www.ncbi.nlm.nih.gov/, PRJNA771942; https://www.ncbi.nlm.nih.gov/, PRJNA771922.

## Author Contributions

JZ designed the research and wrote the original draft of the manuscript. AQ contributed to the data analyses. BW provided technical guidance. XZ and QD conducted the experiments. JL contributed to the experimental-figure-drawing. All authors have read and agreed to the published version of the manuscript.

## Conflict of Interest

The authors declare that the research was conducted in the absence of any commercial or financial relationships that could be construed as a potential conflict of interest.

## Publisher’s Note

All claims expressed in this article are solely those of the authors and do not necessarily represent those of their affiliated organizations, or those of the publisher, the editors and the reviewers. Any product that may be evaluated in this article, or claim that may be made by its manufacturer, is not guaranteed or endorsed by the publisher.

## Footnotes

1
https://www.ncbi.nlm.nih.gov/genome

## Supplementary Material

Supplementary Figure 1 Read length distribution of transcriptome sequences. **(A)** CCS sequence. **(B)** FLNC sequences. **(C)** Consensus isoforms.
