# Supplementary figures and images for: Integrated Analyses of Transcriptome and Chlorophyll Fluorescence Characteristics Reveal the Mechanism Underlying Saline–Alkali Stress Tolerance in Kosteletzkya pentacarpos
Source: Front Plant Sci. 2022 May 6;13:865572. doi: 10.3389/fpls.2022.865572 (PMC9122486; doi:10.3389/fpls.2022.865572)

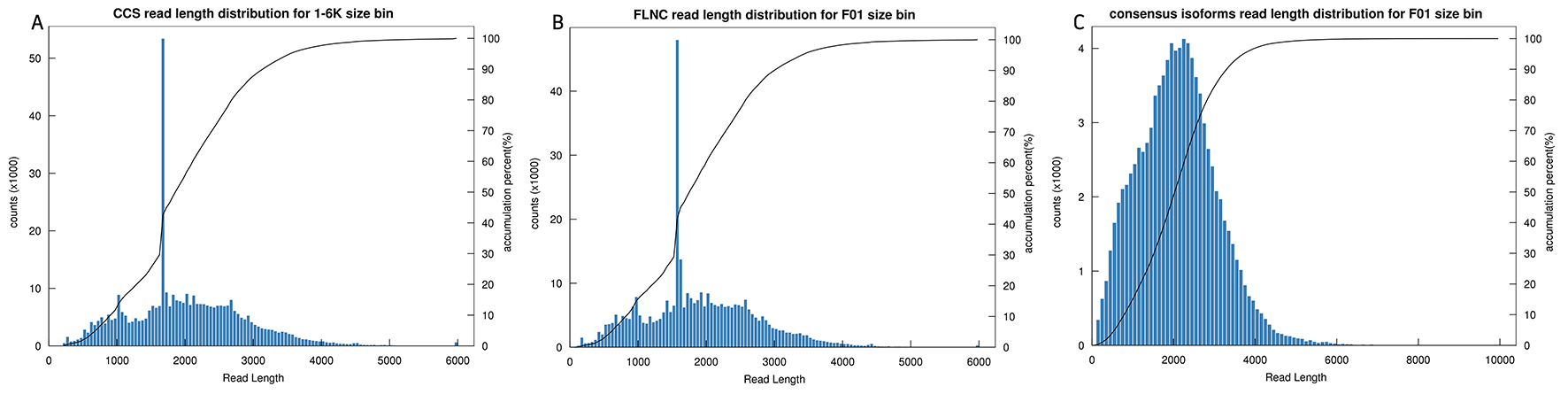

Supplement: Supplementary Figure 1 — Read length distribution of transcriptome sequences. (A) CCS sequence. (B) FLNC sequences. (C) Consensus isoforms. [file Image_1.TIF]
