# Supplementary material for: Integrated Analyses of Transcriptome and Chlorophyll Fluorescence Characteristics Reveal the Mechanism Underlying Saline–Alkali Stress Tolerance in Kosteletzkya pentacarpos
Source: Front Plant Sci. 2022 May 6;13:865572. doi: 10.3389/fpls.2022.865572 (PMC9122486; doi:10.3389/fpls.2022.865572)
Supplement: Supplementary file 2 [file Table_1.DOC]

**Supplementary Table 1.** Core genes from specific modules in *K. virginica* seedlings under salt and alkali stress

| Gene module | Gene ID | kME |
| --- | --- | --- |
| Meblack | F01_transcript_10267 | 0.994183470309566 |
|  | F01_transcript_26643 | 0.990872799112157 |
|  | F01_transcript_38396 | 0.996959756666081 |
|  | F01_transcript_42549 | 0.99076282546198 |
|  | F01_transcript_47548 | 0.989336846529814 |
| Mebrown | F01_transcript_12395 | 0.999060417853016 |
|  | F01_transcript_13494 | 0.99810207810731 |
|  | F01_transcript_20723 | 0.995896242137185 |
|  | F01_transcript_57306 | 0.99472931383993 |
|  | F01_transcript_62089 | 0.99342844229821 |
| Megreen | F01_transcript_13867 | 0.996108054432897 |
|  | F01_transcript_50704 | 0.988624433262301 |
|  | F01_transcript_74832 | 0.993799225304551 |
|  | F01_transcript_91440 | 0.986018505000158 |
|  | F01_transcript_95488 | 0.986181863127749 |
| Memagenta | F01_transcript_13312 | 0.988231162178504 |
|  | F01_transcript_25894 | 0.9962772332107 |
|  | F01_transcript_59507 | 0.993478880823985 |
|  | F01_transcript_7879 | 0.988034576513688 |
|  | F01_transcript_9571 | 0.994751350269247 |
| Meyellow | F01_transcript_3631 | 0.997432661885303 |
|  | F01_transcript_38286 | 0.993765207129276 |
|  | F01_transcript_4187 | 0.998962144316694 |
|  | F01_transcript_487 | 0.994334247992668 |
|  | F01_transcript_9686 | 0.995401212475674 |
