# Supplementary material for: Integrated Analyses of Transcriptome and Chlorophyll Fluorescence Characteristics Reveal the Mechanism Underlying Saline–Alkali Stress Tolerance in Kosteletzkya pentacarpos
Source: Front Plant Sci. 2022 May 6;13:865572. doi: 10.3389/fpls.2022.865572 (PMC9122486; doi:10.3389/fpls.2022.865572)
Supplement: Supplementary file 3 [file Table_2.DOCX]

**Supplementary Table 2.** VIP values of the core genes in OPLS-RA model

| Var ID (Primary) | M2.VIP[1+3+0] |
| --- | --- |
| 9571 | 1.58114 |
| 3631 | 1.45615 |
| 59507 | 1.40846 |
| 53932 | 1.40044 |
| 25894 | 1.35854 |
| 4187 | 1.32629 |
| 7879 | 1.24054 |
| 13312 | 1.21069 |
| 95488 | 1.10532 |
| 62089 | 0.957626 |
| 13867 | 0.951644 |
| 13494 | 0.878585 |
| 487 | 0.866078 |
| 57306 | 0.854767 |
| 20723 | 0.85005 |
| 74832 | 0.805104 |
| 12395 | 0.799802 |
| 91440 | 0.698162 |
| 9686 | 0.670909 |
| 50704 | 0.666658 |
| 38396 | 0.620471 |
| 26643 | 0.501522 |
| 47548 | 0.443416 |
| 42549 | 0.433066 |
| 10267 | 0.339284 |
