# Supplementary material for: Integrated Analyses of Transcriptome and Chlorophyll Fluorescence Characteristics Reveal the Mechanism Underlying Saline–Alkali Stress Tolerance in Kosteletzkya pentacarpos
Source: Front Plant Sci. 2022 May 6;13:865572. doi: 10.3389/fpls.2022.865572 (PMC9122486; doi:10.3389/fpls.2022.865572)
Supplement: Supplementary file 4 [file Table_3.DOCX]

**Supplementary Table 3.** Full-length transcript sequences of key genes in *K. virginica* seedlings under salt and alkali stress

| F01_transcript_53932.cDNA (2715 bp) |
| --- |
| GAGTTAGGGTTTTAGAGGCCTGTAACTTTGCAATCTATTGCGAAATTAAGCATGCGTTCCCTCTGTTGCGGAATTAAAGA  ATTTTTTTAGCTTCTCCAATTTAATTCACTGTTAAAATTCTACTCGAAACGCTCTTTTCGAGTGTATGTTCCTTTAATAG  ATAGAAATATGGCTTCAACGGGGCAGCCACCGTCACTTAAAAAAAGAGATGCGCCATCGACGAGAGAAGGGGATCAACTT  ACTCTTACCCCTTTAGGTGCCGGCAGTGAAGTCGGTCGTTCTTGCGTTCACATAGACTTACAAAAGCAAAACTGTACTGT  TTGATTGTGGAATTCATCCTGGTTACTCGGGTATGGGTGCTTTGCCGTATTTCGATGAGATTGATCCTTCAACAATAGAT  GTTCTTCTTATCACCCAGTATGCCTTTCCCCCCGTTATTTTCTCTTCTTTTCTCGTTCAAATTTGGTGCTGTGTAGAGTT  TAACTGTTGCACATTTCATGTGCTTTCGCCGTATGTTCAGCTTTCACTTGGATCATGCTGCCTCCCTACCATATTTTCTA  GAAAAGGTTTATTTTTTTCAGAGTAAAACTATCGTGTAAGTTTTAATCATGTGGTCTTGTTTAACTAATGTGTTGATATG  TGCAACCAGACCACGTTCAGGGGTCGAGTTTTTATGACCCATGCAACAAAAGCAATTTATAAGCTTCTTCTGACTGATTA  TGTTAAAGTGAGCAAAGTTTCAGTTGATGACATGTTATTTGATGAACAAGACATTAACCGCTCCATGGATAAAATCGAGG  TGTATATATTTTCTTGGTTTTCCATTTCTTCGAATGTTTAGTGCCCTTGTGTCTCAGTGTTGTTAGATTTGAAAATTATG  TGTTGTGTTTTGTGCTTGACTGCAAGGTTATTGATTTCCACCAAACTGTGGAAGTAAATGGTATTAAGTTCTAGTGCTAT  ACTGCTGGGCATGTTCTTGGTGCCGCTATGTTTATGGTTGACATTGCCGGGTGTTCGAGTGCTCTACACTGGAGATTATT  CTCGTGAAGAAGATCGACATCTCCGTGCAGCTGAGCTGCCACAGTTCACTCCTGATATATGTGTAATTGAAGCCACTTAT  GGAGTCCAGCTCCATCAACCACGACATATTCGGGAGAAGCGATTCACTGATGCTATCCATTCTACTCTTTCTCAAGGTGG  TCGTGTTCTAATCCCAGCGTTTGCCCTTGGTCGTGCTCAGGAACTTCTCTTGATCCTTGATGAGTATTGGTCAAACCATC  CCGAGCTTCATAACGTTTCAATATATTATGCTTCCCCACTTGCAAAAAAGTGTATGGCTGTTTATCAGACATACATACTT  TCCATGAATGAGAGAATTCGAAATCAGTTTGCGAACTCAAACCCCTTCAAGTTCAAGCACATTTCTCCATTAAATAGCAT  CGAGGAATTTAATGATGTAGGTCCATCTGTTGTAATGGCAAGTCCAGGTAGTCTTCAGAGTGGGCTGTCACACCAACTCT  TTGACAAGTGTTCTGATAGAAAAATGCTTGTGTTCTACCTGGTTATGTCGTCGAAGGGATAAAACGATTATTAATGAACC  AAAGGAAGTGACACTCATGAATGGTCTCAGCGCTCCTTTAAACATGCAGGTTCATTACATTTCGTTCGCTGCCCATGCAG  ACTATGCTCAGACAAGTACATTCTTGAAAGAGCTAATGCCTTCCAACATAATTCTTGTTCACGGAGAAGCAAATGAGATG  GGAAGACTCAAACAGAAGCTTATCACCGAGTTTACTGATGGCAACACCAAAATCATTACCCCTAAGAATTGTCAGTCTGT  TGAGATGTATTTTAGCTCCGAAAAAATGGCCAAGACCATCGGAAGATTAGCTGAAAAGACCCCAGAAGTTGGTGAAACTG  TTAGTGGTGTTCTGGTAAAGAAAGGTTTCACCTACCAGATAATGGCTCCCGATGATCTCCATATATTCTCACAGCTTTCA  ACTGCAAACATCACTCAGAGGATTACTATTCCGTTCACTGGTGCATTTGGTGTGATAAAGCATCGACTTGAGCCGATATA  CGAGAGTGTGGAATCTTCAACAGATGAAGAATCTGGGGTTCCAACATTGCTAGTACACGATAGAAGTGACTATGAAGCAG  GATTCAGATAAACACATCTCATTGCATTGGACATCAGATCCCATAAGTGACATGGTGTCGGATTCCATCGTGGCATTGGT  TCTGAACATTAGCCGGGAGACCCCTAAGGTAGTAGTTGAGTCTGAGGCCGTAAAAACGGAAGAAGAAAATGGGAAGAAGG  CAGACAAGGTTATTCATGCTCTCCTGGTTTCACTTTTTGGGGATGTCAAATTAGGAGAAAACGGAAAGCTTGTGATAACT  GTTGATGGGAATGTGGTTCATCTTGATAAACAGAGTGGGGACGTCGAGAGTGAAAACGAAGGTCTAAAGGACAGAGTAAA  GACGGCTTTTCGACGAATTCAAGATGCAGTAAAACCAATCCCTCCCTCAGCATCATAGTTTTATCAATCCTTCAATATCT  TCTGATTCACTGGTTCATCTTTTAATGTACATGAGTTTATATTTATGGTGTATGATAATATGTTGTATTCATTTTCTTTT  TCTCTTCGTGTTCATTTGATTCGCTGCTGTTACTTCTAAATTCACTTGTTTTTCTTATACAAACTGAAAATTTCC |
| F01_transcript_13312. cDNA (2230 bp) |
| GAAATATAAAAAGAGGAGAGAAGAAGAAACCTAGGAAGCACAGCGCCTGGCTTGGGTTCCTCCTTGGTGTGTGTGTGTGT  GTTCGGCGGTTCTCTCGTGCGTTTCACGTGTTAGATCCGTCTTCCCAATCCCTACATTCTTCCCTAACCAACCCTAATTC  CCTTCTCCTCTCTCCCCCAATTTAAACATCACTCTCCTGTATCTCACACCATTGATTCTCCCTTCTCCCCCAAAGCTTTG  ATTTGGACTTTACAAAAACACAATCCGATTTCTTTGAATGGCTATGACGGCCGATTCTTCTTCCCCGCGCGACGGTCTCA  AGAGCCCTCAGTTCCGCCACAAGAATTCTCCTTCTAAACAGCCTTGGTCTCAAGTGGTTGCTGCCCATCACTCTCCTCCT  TCTCCCGTCGCTTCCCTGCCGGGGCAATCAAGTTTTTCCGATTGCCCCCCTTCGCAACCGCCTTCTTCCTCTTCCTCTTC  TCCGCCTCCGCCGCAGGGTATTTCTTTAGCCGCCGCTGAAGGTGGTTCTGATTCTAATACCAACAACATTACTTCTCCAA  GCTCCAAGAAGCCGGCTTGGAACAATCCTTCAAACGGCGTCGTCGTTGGGGTGGATTCTGAAACGGAAGACGGGTGGCCT  GTTTTATCTAAATCCGGTAAGGGTCTCCAAAGATCATTACCCGGCTCTTCTTCCAGGAATATTGTTGTCGGATCGTTTTC  TACTTCTCAGGTTCCGGTGGTTCTCCAATCAACTCAGAGACAAGGTACAAATAATGCTAACCCCAATTCAACACCGAACC  GCCCAATGGTAAATCGGCAAAAGCCTTCTAAACGCAGTGGCAATAGCTCTGGTACTGGCAATATCTCCGGCAGTGGCAAT  ATCTCCATCAGTGGCAATAGCTCCGGTGCTGGCAATAGCCCTGGTGGTGCCAATATCTCCGGTACTGGCAATAGCCCTGG  CGGTGGCAATATCTCCGGTAGTGGGCCACCACCACTGCCATTTACCGAGCCACAGATGTCTCCAACTAATGTTGTTCCAA  CAATGCCATATCGTATACCAAGAGACCCCCGGTTCAGGGGCAATAATTGGGAAACCAGACCAGTTGGAGGCTTTGCATCA  CAGTCACACAATGATCATCGGCATTCTTCTCGAAGGGGAAGTAACTATGGACCACGTGGAGATGGTGGCTATAACAATTG  GGGGAGGCGTGATCAGGACCGTGGAAATTATGGGAATGCTAGAGATGGTTATATGCAACCTATGAGAGCACCCCTAAGGG  GCTTCCCAAGACCTTCACCTCATGCGGTTCATCCTTCTGTTCGTCCTCAACCTGTGGATCCATTTATGAATCCCACAAGA  GGATATCCTGAGTGTATGTATTTGACTATGGAGCAATTTATGGGCATGACAGCATTTGCCCCTGCCCAGCCTACAATATA  TGTGCCTGTTCCAGATCCTCCTCTGTCTGCGTTGTTATTACATCAAATTGATTATTATTTCAGTAATGATAATTTGGTTA  AAGATGACTATCTGAAGTCTAATATGGATGATCAGGGTTGGGTAGCCATTTCTTTAATAGCAGGATTTCCCAGAGTTAAG  AGTTTGACAAGCAACATTCAGCTGATAGTGGATTCATTACGAAGTTCAACTATTGTGGAAGTAGAAGATGATAAAGTGAG  GAGGCGTAATGATTGGAGGAAATGGATACCTTATCGGATTTCAACAGTGCCGGGATCAATGTCTCCAGGCGGATCAAGTC  CTGAAGTGCTGGCAAGTTCTTTTCAGCAGGTTACGATTAGGAAAGAGTCTACCCTTGCTGAGGATACTTCAGGAAGACAT  CCGTCACTCTCTAACGCTGAAGGTTCAGAAGACCGTTGTCTGGACAAAAACTGATCAATCTCTGCCGCTACAAAGCACAT  TCCGGTGAGGAAGCTGTGCTTTATTTATTTTTTGATTCGAGTGAAGTGGAGGCTGCATCTGAAAGAATTGGACACCTTTT  TTTTCTTTTATTTTCGCCCATCACCTTATGTGGTTTTGACGGTGATATGGTGTGGCGATTAGAGGCAAAAAATAAGATAC  ATAAAATTATCTTTTGAAACATAGAGAAAGTGAAGAAACATTGGAAAAAGAAAAGAAAGAGTTGACTGAGGAAGAATAAT  GAGGCCTTTTGGTTGTACAGTTTTGCTATTATTATTATTATTATTATTATTATTATTATTACTTATTGTC |
| F01_transcript_3631.cDNA (3170 bp) |
| GATTAAAGTTTTAATTTCAGTCAGTCCTTAATTTTGAGCATTTCGAGTGAGAGGAAGAGCAGAAAGAGAGAGAGAAGCTT  GAAATTGATAATTTCTTGCAGGAATTAGGGCTTCTACAGTTGTTTTCTCTACGAATTTCAGATCTGATTTTCTAGATATG  GACTCGGTTGAGCAAGATGGAAATGGGGCGGTGCCCGAGTCTCTGCCTCCTCCTCCACCTGTGCCTCCGGATGTGGTTCC  AATCAAAGCAGAACCTGAACCCGCAAAGAAAAAGGTTGTGCGGGTCCCAATGGCCAGGCGTGGTCTTGGGTCTAAGGGGC  AGAAGATAACTCTTCTCACCAATCACTTCAAAGTGAATGTTGGAAGTGTTGATGGATATTTCTTCCACTACTGTGTTTCT  CTGTTTTATGAAGATGGCCGTCCAGTTGATGGCAAGGGTGTTGGACGAAAAGTGATAGATAGAGTGCAAGAAACCTATAG  CAATGAGTTGGCGGGAAAGAATTTTGCATATGATGGGGAAAAGAGTTTGTTTACTGTTGGTCCTCTCCCAAATAACAAGC  ATGAGTTTACAGTTGTTCTTGAGGATGTTACATCTAATAGAAACAATGGGAATGCAAGTCCTGAAGGCCATGAGAGCCCA  AATGCTCATGACAGGAAGAGAATTAAACGGTCTTATCAGTCAAAAACCTTTAAAGTGGAGATTAGTTTTGCTGCAAAGAT  TCCCATGCAGGCCATTCAAAATGCTCTGCGTGGACAGGAATCCGAAAACTCGCAAGAAGCCTTAAGGGTGCTAGATATCA  TTTTACGGCAGCATGCTGCAAAGCAGGGATGCCTTCTTGTTCGCCAATCTTTCTTTCAAAATAATCCCGACAATTTTACG  GACATTGGAGGAGGTGTCCTAGGCTGTAGGGGGTTCCATTCTAGTTTCAGAGCCTCTCAAGGAGGCCTTTCCCTGAACAT  TGATGTATCAACTACTATGATAATTAGACCCGGTCCAGTTGTGGATTTTTTACTTGCCAATCAGAATGCCAGAGATCCTT  ACTCCCTGGACTGGACCAAGGCTAAACGTACCCTTAAGAATCTGAGGATACAAGTCAGTCCATCCAATCAGGAGTACAAG  ATTACTGGATTGAGCGAGCAAATGTGCAAAGATCAGTTGTTTTCTTTGAAGCAGAAAAGTATGAAGAATGATAATGGTGA  AGCTGAGAATATAGAGATTACTGTTTATGATTATTTTGTAAACCATCGCAACATAGAGTTGCGTTATTCTGCAGACCTAC  CATGCATTAATGTTGGAAAGCCAAAACGGCCAACTTATATTCCTTTGGAGCTTTGTTCCCTGGTGTCTTTGCAACGTTAC  ACCAAAGCACTAACCACTTTCCAGAGAGCTTCATTGGTTGAAAAATCAAGACAGAAGCCTCAGGAAAGAATGACTGTTTT  GTCTAGTGCGTTACAGAGGAGCAATTATAGTGCTGAGCCAATGCTGCGTTCATGTGGTGTTTCTATCAGCACTGGTTTCA  CTCAAGTGGAAGGCCGTGTTCTGCCTGCTCCAAGGTTAAAAGTGGGGAATGGTGAAGATTTCTTTCCACGGAATGGGCGA  TGGAATTTTAACAACAAGAAACTAGTGGAGCCAGCCAAAATTGAACGATGGGCTGTTGTTAACTTCTCGGCACGATGTGA  TACAAATAGCCTTGTCCGGGATCTGACTAGATGTGCCGATATGAAAGGAATTCGGATAGACCCTCCATTTGATGTGTTTC  AAGAGTCGAATCAGAACAGACGCTGCTCTCCTGTTGTTAGAGTTGAAAAAATGTTCGAGGAGATCCAATCAAAACTTCCT  GGAGCTCCTCAGTTCCTACTTTGTCTTTTGCCTGATAGGAAAAATTCTGATCTTTATGGGCCATGGAAGAAGAAGAATCT  TGCTGAGTTTGGTATAGTCACGCAGTGTATGGCTCCAACTAGGGTCAATGATCAGTACCTGTCAAATCTGATTCTAAAAA  TCAATGCAAAGCTTGGAGGACTGAATTCGATGTTAGCAATTGAGCAAACACCTTCAATTCCAGTTGTTTCCAAGGCTCCA  ACCATCATCCTTGGCATGGATGTTTCTCATGGCTCTCCTGGGCAGTCTGATATTCCATCAATTGCTGCGGTGGTCAGCTC  CAGGCAGTGGCCTTTGATTTCCCGCTATAGGGCATCTGTGCGGACACAGTCACCTAAGCTTGAAATGATAGATTCTCTGT  TCAAACGAGTGTCTGACAAGGAGGATGAAGGTATCATTAGGGAGACTCTCTTGGACTTCTATACAAGTTCAGGAAAGAGA  AAGCCTGATCAAATCATAATATTTAGGGATGGGGTTAGTGAGTCGCAGTTCAATCAAGTTTTGAACATTGAACTGGATCA  AGTCATCGAGGCTTGCAAATTTCTTGATGAGAGCTGGAACCCCAAGTTTGTGGTCATCGTTGCACAGAAAAACCATCATA  CAAAGTTTTTCCAGCAGGGATCTCCTGACAATGTCCCACCCGGCACTGTTATAGACAACAAAATCTGTCATCCCAAGAAC  AATGATTTCTATCTTTGTGCTCATGCGGGAATGATTGGAACCACAAGGCCTACTCATTACCATGTCCTCTTAGATCAGAT  TGGGTTTTCAGCTGATGATCTCCAGGAACTTGTACATTCTCTGTCATACGTGTATCAAAGGAGCACCACTGCCATATCTG  TAGTTGCTCCAATATGCTATGCACATTTGGCAGCCTCACAGGTGGGGACATTCATGAAGTTCGAAGATGCCTCGGAGACA  TCCTCGAGCCATGGTGGGGTTACTGCCCCCGGAGCTGTTTCTGTCCCTCAGCTGCCTAAGTTGAAGGACAGCGTGTGTAA  CTCTATGTTCTTCTGCTGAGAGGGACGTATGAGCCCCATGTTTTGTATAGGTATTCGGTTTTACGCCGGAGGACTGAAAC  GGTTTTAAACAAGTTTGTTAAGTTAAGAGTAGGGTAATCCCTGAATGTTTATGATGATGATGCAGTGTGGCTTGAATTGA  AGCATCATCCGTTTTGAGTATGAACCTTTTGGGTGTGTACCCTACTGTATCTACTCTAGGATTGTGAATGTTTGTCGGAC  TAGAACTTGAACTTCTCCCGCCTGTTTAATTAGTACTTGGTTTTAATGCT |
| F01_transcript_7879.cDNA (2664 bp) |
| GGAAGCGCAGAAAGCGAGGAAATCAGGATTCAGAAAAGATAAAAACAACAGGGACTATAAAGATGATCGCAGACGGAGTT  GAAGACGAAGAGAAATGGCTCGCCGCCGGCATTGCCGGCCTCCAGCAGAACGCTTTCTACATGCATCGCGCTTTGGATTC  GAACAATCTCAGAGATGCTTTGAAGTACTCTGCTCAAATGCTATCAGAACTCCGAACCTCAAGGCTTTCCCCTCACAAAT  ACTACGAATTATATATGCGAGCATTTGATGAATTGAGGAAGCTGGAGGTGTTTTTTAAGGAAGAGACAAGGCGTGGTTGC  TCTATTGTCGATCTGTACGAGCTAGTACAGCATGCTGGCAATATATTGCCTAGATTGTATCTCCTTTGTACCGTAGGATC  TGTTTATATCAAATCTAAGGAAGCTCCTGCTAAGGATGTTCTTAAAGATCTTGTTGAAATGTGCCGTGGAATTCAGCATC  CTGTACGTGGCCTCTTTCTTAGGAGTTACCTGGCTCAAGTTAGCAGGGATAAATTGCCTGACATAGGTTCTGAGTATGAA  GGGGATGCTGACACCGTTGTGGATGCTGTGGAATTTGTGCTGCAAAACTTTACAGAGATGAACAAGTTGTGGGTGCGGAT  GCAGCATCAGGGACCTGCCCGAGAAAAGGAGAAACGGGAGAAAGAGAGGAGCGAACTAAGAGATCTTGTTGGGAAGAATC  TTCATGTGCTCAGTCAGATAGAGGGTATTGACCTTGATATGTACAAAGATACTGTTCTTCCGCGTGTACTTGAACAGGTT  GTCAATTGTAAAGATGAGATAGCTCAGTACTATTTAATGGATTGCATAATTCAAGTCTTTCCTGATGAGTACCACTTGCA  AACTCTTGATGTATTGTTGGGTGCTTTCCCACAACTTCAGCCAGCTGTTGACATTAAAACTGTGCTATCTCGATTAATGG  AAAGGCTATCAAACTATGCTGCTTCAAGTGCAGATGTGTTGCCTGAGTTCTTACAAGTAGAAGCTTTTGCAAAGTTGAAT  AATGCCATTGGAAAGGTGATTGAAGCACAACCTGATATGCCTATCCTTGGTGTGATAACTTTATACTCGTCTCTTCTTAC  TTTTACTCTCCATGTTCATCCGGACCGGCTTGATTATGCTGATCAAGTTTTGGGAGCATGTGTTAAAAAACTCTCTAGCA  AAGGAAAGCTTGAAGACAACAAAGCAACAAAACAGATTGTTGCACTCTTGAGTGCTCCACTTGACAAATATAATGACATT  GTTACAGCATTGAAGCTTTCAAATTATCCTCGTGTTTTAGAATACCTTGACGCTGAAACAAATAAAGTCATGGCAACTGT  GATAATTCAAAGCATTATGAAGAACAAAACTCGTGTCTCTACTGCTGACAGGGTTGAGGCATTGTTTGAATTGATAAAAG  GTCTTATTAAGGATCTGGATGGGGATGCTCATGATGAGGTTGACGAAGATGATTTCAAGGAGGAGCAGAATTCTGTAGCA  CGCCTTATTCAGTTGTTGCATAATGATGACCCTGAGGAGATGTTTAAGATAATTTGCACGGTTAGGAAGCATATCTTGGG  TGGAGGACCAAAACGCCTACCCTTCACTGTCCCTCCCCTTGTTTTCTCTTCGCTTAAGTTGGCTCGGCAGCTACAAGGCC  AAGAGGAAAATCCTTTTGGAGAGGAGGAATCAACAACGCCCAAGAAAATTTTCCAGCTTTTGAATCAGATTGTTGAAGCT  CTGTGTAACATTCCAGCTCCTGAGTTGGCACTACAGTTGTATCTACAGTGTGCTGAGGCTGCTAATGACTGTGATTTGGA  ACCTGTGGCATATGAATTTTTCACACAAGCATATATTTTATACGAAGAAGAGATATCGGACTCAAGAGCACAAATTACTG  CAATACATTTGATAATTGGAACGTTGCAAAAGATGCATGTCTTTGGTGTTGAAAATAGAGATACCTTAACTCATAAGGCT  ACAGGGTATTCCGCAAAACTTTTGAAGAAGCCTGATCAGTGCAGAGCTGTTTATGCATGCTCACATCTCTTCTGGGTTGA  TGATCAGGACAATGTGAAAGATGGAGAGAGGGTCCTGCTTTGCCTTAAGCGGGCACTGAGAATTGCAAATGCTGCTCAAC  AAATGTCGAATGCAGCACGAGGTAGCACTGGATCGGTCACTCTCTTTGTTGAGATACTGAACAAGTACCTTTATTTCTTT  GAGAAGGGGAACCCCCAGATCAATGTAGCCGCGATCCAGAGTCTTCTTGAATTGGTTACAACTGAGATGCAGAGCGATTC  TAGTACACCGGATCCAGCGGCAGATGCTTTCTTTGCCAGCACGCTCAGATACATAGAGTTTCAGAAACAGAAAGGTGGAG  CGGTAGGTGAAAAATATGAGCCCATCAAGGTGTGACCGCGAGAGCTCCTTTAAAATATGTAGACGTTTGATTTGATTTGT  ATTGTGCAAAAGGGAAAGTGGATTTATAGTACATGCTGGAGTTTCAATTGTTATCGCTGTTGTCCTACTCCTACATTTTT  TCGGTATAAAAAATTATGTACGCCGATCAGATCGTATCACGTGGCTCGTTTGAGGAAAAGACCACCTGACCTATAATTAA  AAAGGGTAAATTTCATGTTAAGTG |
| F01_transcript_59507.cDNA (3492 bp) |
| GGGAAATAACAGAGTTGGAAACTGATCCTCAAAAACGGAGAAAAAGTGCAATAATCTTACTGGGAAAATGGAGGTCGTCG  ATCAATCCTCAGTCTTCATAGTATTTATCTAAAACTTATTTCCTCATTCAATTTTGCTTTACTTGTCCCGTTTTTAGTTC  GTCCCCATCACTAACTACAGACAACGGCTCCCTTTTACTAAAATCCCCCCTTCGTTTTTAGCTCCTCGGTCAAAGATGTG  CGGGGAAACTATATTTGCTGTAGATTTTTGGTGAGTTTAGTTGAGAATTTTGTGTGTATGGGTTTGTTTGTGATGTTTTA  TTGATTCGAGCATGGCCGTGGTGAGCGTTGCGCCCGTAATAACTCAAGGTGTGAATTCGATGAGGGTTCGTGACGTTAGA  GGTCCAGGGATCATGTCGAGTCCAAGAGAAATCGTGGAGGAAATGGTGGAGGATATAATTGAAGAGAAAATCTATGTCGC  GGTTGGGAAAGATGTGGAAAAGTATAAATCCGTTTTGATCTGGGCTTTACAACATTCGGGAGGGAAAAAGATTTGCATAA  TTCATGTTCACCAACCGGCTACTATGATCCCGGTTGCTGAAATGGGTACAAAATTTCCAGCTAGTAAACTCAAAGAACAA  CAAGTCAAGGCACACTGGGAACTTGAAAGAAAACAAATGCAGGACATCCTGAATGAGTACCGTTTGATTTGTCTCCAACG  TGGGGTGCAAGCAGAGAAGCTGTACATTGAAATGGATTCTATTGAGAAAGGGATTTTGGAAATGATATCTAAGAATGGGA  TACACATGCTAGTAATGGGAGGAGCTGCAGACAAACATTATTCAAAGTATAAACCACATAAGAAAGCTGTTGACCTCAAG  TCCAAGAAAGCCATCTTTGTGCGTGAAAATGCACCTACTACCTGCCACACAATATGGTTTCTCTGCAAGGGGCTTCTTAT  CTACACGAGGAATACAAGTTCAGATGTAACTGATAAAAAGGTTGCAGCATCATTGCCAGCAAGTCCAGACCTTGGATCTG  GGCAAAATCATATCAGGTCACAATCTGTGATATTGCGACCAACTAGTAAAGTAAAACCCTCTACTTCTGCACTAGATTCG  TTGTGCAGAGCGAGATCTGCAAATGTTCATGGGCGTGTGGGAAGTTCACTATATTTTACTTCTCCTGATGGTAATGAAAG  GATATCAACTCCACAAAGAAGATCTGATGTAGAAGGGATTTCTGATGAATGTGATAGTTTATCCGGAAGAAGTCCTCAGA  GTTCAGTTTTTTCACCATGTTCTTCCAGTGGAATGGTTGATGCAGCTTTAGTCCCAAATGAAAATGGATTGCAATATAGT  TTCATTCCTCAGGCCGAACGGAATTTTAATCTATCATCTCTTCCCGGTGTGCTGGATGGAACCGATAGTACTCTTTATGA  CCAACTCCAACAAGTAATGGCCGAGGCAACAAACACAAGAAGAGAAGCTTTTGAAGAGGCAATGAAGCGTGCAAAAGCAG  AGAAAGATGCTTTTGAGGCTATACGCAGGGTCAAAGCATCAGAAAACCTGTATGCACAGGAGTTAAAGCAAAGGAAAGAA  ATTGAGGAAGCTTTAGCAAAAGAAAAAGTGGAACTTGGCAAGATGAAGAACCAACGGGATGAGGTCATGATAGAACTTCA  AGCTGCCTTGGATCAGAAATCATCGCTGGAGACACAAATTGCGGAATCTGAAAAGGAGGTTGAGGAGCTTGAAGAGAAGA  TACTCTCAGCCGTTGAACTTTGCAGAATTACAAGAAAGAAAGAGAGGAGCTGCAGATGGAGCGAGACAATGCACTCAAAG  AGGCTGAAGAGTTGCGGCAAAGTCGAGCTGAGTCCTCAGGTGCACATATGCATCAGTTTTTCACTGAGTTCTCTTTCGCA  GAGATTGAGGAAGCAACTCTCAACTTTAGTCCCTCCTTGAAGATTGGGGAAGGGGGATATGGAAGTATTTATAAGGGTAT  TCTACGTCATATCACTGTGGCCATAAAGCTGTTGCATTCTAATAGTTTGCAAGGACCATCGGAATTTCAACAAGAGGTCG  ATGTTTTGAGTAAGATGAGGCATCCAAATATGGTCACTCTCATTGGAGCCTGTCCTGAAGCTTGGACTCTTGTTTATGAA  TATCTTCCCAATGGAAGTCTTGAAGACCGACTTAGTTGCAGAGATAATTCCCCACCTTTATCTTGGCAAACACGTATCCG  TATCGCTACCGAGTTATGTTCTGTGCTTATATTCCTTCATTCTAGTAAGCCTCATGGCATAGTGCATGGAGATTTGAAAC  CTGCAAACATTCTACTTGATGCCAATTTTGTTACTAAACTGAGTGACTTTGGAATCTGTCGCCTCTTAAGCAACAATACA  ACTGTCTGCTGTAGAACTGACCCCAAGGGAACTTTTGCGTACATGGATCCAGAGTTCCTTTCAACAGGAGAACTTACTCC  AAAAGCAGATGTTTACTCATTTGGAGTCATTTTGCTACGGTTGTTGACTGGAAAACCAGCCTTTGGTATAATAAAGGAAA  TGCAATATGCATTAGATAATGGAAACTTGAAGCAACTCTTGGATCCTTTGGCCGGAGATTGGCCTTTTGTGCAAGCTGAG  CAATTGGCTAACCTAGCTTTGAGGTGCTGTGAAATGAACCGGAAGTGTCGACCAAACCTTTCAACAGATGTGTGGAGGGT  GCTTGAACCAATGAGGGCTTCGTGTGGAGGCTCATCCTCATTTCAGCTGGGTTCTGAAGAGCATTGCCAACCTCCTCCTT  ATTTTATCTGCCCCATCTTCCAGGAAGTAATGCTAGACCCTCACGTAGCATCAGATGGTTTCACTTATGAAGCAGAGGCT  GTGAGAGGCTGGCTTGATAGTGGTCATGACACTTCACCCATGACAAACGATAAGTTGGAACATTCCAATCTAGTCCCTAA  CCTTGCTCTTCGCTCTGCGATTCAAGAGTGGTTGCAACAACATTAAAGTGCAGTACTATCATTTCATCATTTAGCTTCAA  TATATTGTTCACCGTTTATAATTGTTTCTTAAGTATCCAAAAGCTAGAAAAACATCAGAATTTGTCTATTTGTATTTTCA  TATTCCTCTTTTTTTTTTTCTTGGGTATATGGTGATTTGTTCTGTAAATTTGGGGATGGAAACCAATTGATGAGGTAGGC  AGCTCGTTGACATGCAAATGTTCAAGGATGATGCTGGCGATTGGACTCCGTGCAGGCTTTTGTTTCTCCGGCAATCGTGT  CATCCATTATATGCATACAGGAGATTGTTCGCATTCCATGGGATTCGTAGGTGAATATTTACAAAAATAAGTCTGTTATG  GGCGTTTTCAATTTTATATATGTGTAAGATTACACTCTTATTCTCATATCTGAAATATGCATTAGGAGTTATAATCACAT  AGTTTGTAGCTACATTTCGACGATTGAATGCTTGGTTTCAAATATTAATCCC |
| F01_transcript_25894.cDNA (1115 bp) |
| GGTCCATCTCTTTCTTTCCCCTTCTGCTCTGCCCTTGCTGTTGGAGTCGCAGAGAAAAGAGAAAATGGGTAATCTACTCT  GTTGTGTACAAGTTGACCAGTCTACAGTAGCCATCATGGAGAGATTTGGTAGGTTTGAGGAAGTGCTTGAACCCGGATGC  CATTGCCTACCTTGGTTTCTTGGAAGCCAACTCGCCGGCCACCTCTCGCTACGGTTGCAGCAGTTGGATGTTCGTTGCGA  GACCAAGACTAAGGACAATGTATTTGTCAATGTTGTTGCATCCATTCAATACCGGGCACTTGCGGACAAGGCGAATGATG  CCTTTTACAAACTGACAAACACAAGGACACAAATTCAAGCTTATGTTTTTGATGTTATTAGAGCAAGTGTTCCAAAGCTA  AATCTGGATGATGTTTTCGAACAAAAGAACGAAATTGCTAAAGCCGTCGAAGAGGAACTCGAGAAGGCTATGTCTGCCTA  TGGGTATGAGATTGTTCAAACACTTATCGTGGACATCGAACCCGACGAGCACGTGAAGCGTGCAATGAACGAAATCAACG  CCGCCGCAAGGTTGAGGGTGGCAGCTAATGAGAAGGCAGAGGCTGAGAAAATTCTGCAAATCAAACGAGCTGAAGGTGAA  GCCGAGTCCAAGTATTTGTCAGGGCTGGGAATTGCTCGCCAACGACAAGCGATTGTCGATGGACTGCGAGACAGCGTGCT  TGGATTCTCCGTTAATGTCCCCGGGACTACCGCAAAGGATGTCATGGACATGGTCCTGGTCACTCAATACTTTGACACCA  TGAAAGAAATTGGTGCTGCTTCGAAATCCTCGGCCGTGTTCATCCCTCATGGTCCGGGGGCTGTTCGCGATGTTGCTACT  CAGATTCGTGATGGACTCCTCCAGGCTTCACAGCAGTAACTATAGCACATTTCAAAGGTTTATACCAAGTTCTTAAGACC  AGACAAAGATTTATTATTATTATTATTAAGGATATAATATTTGGTTTTCATGTTAAGTTCTTGGCGCCTTCACTCTTGTT  TATTTGTAATATTTTATATAAAGGGAAGTGTACATTAATGAGTGAAATAGGAGGAAAATGCCTATTGTTCCTGCC |
| F01_transcript_95488.cDNA (1331 bp) |
| ATACTGCCCTCTTTGTATTCGTCTTCCCCAAATTTTCAGAGTTCCAACCGTAACAAAGCCAAGCCAACCTCTGTTTGGTA  ATATTTCGAAAACACTGCCATGGCTATGCAAACTGGAGTAGGCTTTTCGAAGATCTTGATCCTAGCTGGTGCAGGTTACA  CCGGTACGATCCTCCTCAATAACGGTAAATTGTCGGATATGTTGGGTGAATTGCAGTCACTGGTGAAGGGACTGGAAAAA  TCTGGGGAGCAAGCTGATGATTCAGATGCCCTTCTGGCTCAGGTGCGTCGTTTGTCAACCGAGATCAGGCAATTGGCCTC  AGCACGACAGATAACTGTTTTGAATGGGGATTCCGGCGGTATGTTGAGCCCTTTTGTTATACCAGCTGCTACATTGGGGG  CATTGGGTTATGGTTACATGTGGTGGAAGGGCATTTCATTCTCTGATCTTTTATGGGTCACGAAACGCAATATGGCAATG  GCTGTGGAGAACTTAACGAAACATCTGGATTCTGTCTCAGATGCTCTCTCTGCTGCAAAGAAGCATTTGACACAACGTAT  TCAGAACTTGGATGATAAAATGGAAACACAGAAGGAAATCTCAAAGAGCATTCAAGCAAGTGTAGAGGATGCTCGTATGA  ATCTTTCTAACATTGAGTACGACTTGGATGCACTGCAGAGAATGATTTCTGGTTTGGATGGCAAAATTGGTTCATTGGAA  TGTAAGCAGGATCTTGCCAATGCTGGCGTGTGGTACCTTTGTAATATGGTTGGTGGCAAGAAAGCAAACATGCCTGAAGC  TCTGCAGGAGCAACTTAAACTTTCTGGTAAATCTCGTGCTTTGCTTACATCAGGAACTCCAACTCCGAAGGGTCTGAAAG  GCTTTGCAGATATTTTATCCGGAAGTGAGAAGGATTCTGGAAGAGATGCTATTGTGAAAGATGGTATTGATAATTTGGAT  GAAGAGCCGAGAAGCCTGCACAGGAGCATTTCAGCTAGGTGTTGATAGTCATCATTTTGGAGTGCTGCAATTGAGAGAAA  TTACATATGCCTATGAACAACATAATTGACGGCTTTCTGCAGGCAGTGCAAATTTGTAGTCTGCGGTTGATGCCTTACAT  ATGAGAATGTAAAATTTGGAGGGGGAAAAACCTACATTTCAGTTTGCAACTACTATGTATTTTGATTGATTCATATGGTC  AGTTTGAGCAATTCGACTTTTGATTAAAAAAATATATAAATTGTAAAGTATGACTTGTAAATTCGACTCACCCAAAAGAA  AGTTCACTCGATTCAATTGAATTGTCCAGCCCTGACTCGGTTGTGTGGCAC |
| F01_transcript_9571.cDNA (2502 bp) |
| GGTAATTTACAAACATCATACTTTTCTTGTCCGATTTGAGCAGATGCTGGAGTTTGAACTCAGCTCAAGCCCAACCAAAA  CCCACATACGCCAGCCTCGTCGCTTTCGACTGTTCATCCTCCGTTCGTCAAGGAAAAAATGATTAAATAAAAAAGCTGCC  AAACAAAGTCAGGATCTTAGATAAACAAACAAACATTTTCAATACCCAGAAAAGAAACTCTTCGGAAGTTGAACGGAGAC  GAAGAAAAACGATGAAATCTCGATTCATCTGAAAGTTTGGAACATAGATTGAGAGGGAGATATGGATCCGGAACAAACGT  TTATAAGGGTTCAGGAGAGATTTTCTCAGATATTAACACCCAGAGTGAGAGTAGCTTTGGAGTACATCTATCTCTTTATT  GCCATTACTTCCTTTTCTATTCTTGTTGTCATGCACGCCAATTATGTTCAACAGCCTGGCTGTTCGAGTGAGCTCTCTGG  AGTTGAATCAAACGAAGTCCAACTTGTTCAGATTAAGATAACCAGTGCTGGCTTGTGGTCACAAGATGAGTCTGAACCCA  CTGTTGTAAATAATCCTGATCCGGAAACTGTAACTGAAAATATAGAAGTTGCAAATGCTGTTGATGATGGGTTGATGTTC  TTGGATGCTAAATTTTGGTTGAGCTGGTTTGGTTCCAGTGCTAGGAGGGGAAAATTGGCATTGAAGTTCTGGAAGACGGA  TGATGAATTTATTGAGAAGCAAGCAGAAAGCTCTACCAATGGTGAAAGCTCTAAGCCAACTATTGCTGATGCTGTTCTTA  AAACAGAGAAAGAGGAGACACGTAGTAGTTTTTATTTATCCGCAAAACAGACATTTAAAGCAGCTTTTCTACACTTTGGC  AAAAAGTGGCACAGACGTTTAGCTTTAGTCTGGAGATATGGCGTTTGGATCGTTGGAAGTTTTCAGAAGTTGTGGAATAT  AACAGGTTTACATTTTAATCTCGATGTTCCTAAATGGTTGCATATACTTCACTTGGATAGGCTTAACATATATGCAGTGC  ACTGGCTTGAGAGAAGAAGTAAAGCATTTGAACCGACTTATCTCTATACCATGGAAAAGGGTTATTTCTTGCTGCCTGAA  GCAGCCAAGTCACGGCATAACATACGTACTATTAATATTAGCATATCAGCTCGACATCCCTGTTTTGGGAACAGGTGGCA  GCAACTTCTCATCAATAGATTTGTGGGGTATGACACTATTTTGATGAATAGTTTATTGCACCAGCCTGGTCAAGGTTATC  TTTATAATTTTCAGACAAAGGAATTCTACAATCTTAGTTATGCTCAAGAATTGCCACATGGCTCCACAAGAGTTGGAGAC  TATCTTGTCACCAAGTGTGGTGTGCTCATGATGTCTTTGTTTGTATTTTTCACAACCACCATGTCAGTATCATTTACATT  GAGGGAAACTCAGACTCGCATGCTGAAGTTCACAGTGCAGCTTCAACACCATGCTCGACATAGGCTTCCAACATTTCAGT  TGATATTTGTGCATGTGATCGAATCGCTTGTATTTGTACCAATTATGATTGGCATCCTATTTTTTCTATTCGAGTTCTAT  GATGATCAGCTTTTGGCTTTCATGGTTCTAATTCTTGTCTGGTTGTGTGAATTATTTATACTGATCAGTGTTCGGACTCC  GATATCGATGAAGTTTTTTCCACGCTTCTTTTTGCTCTACTTTCTGGTTTTTCACATTTACTTCTTCTGCTATGCATATG  GCTTTTCGTATTTGGCACTCTCGACTACTGCAGCATTCATGCAGCACCTTATTCTGTACTTCTGGAATCGTTTCGAGGTA  CCTGCACTTCAAAGGTTTATGCAGAATAGGCGGTCTCAGCTTCAGCAACCTCCGGATTTTCACATCACATCCTCAACTAT  ACTTGCGTCTACACTACACATTACACGATTGAACACCAGGAGTCCCGGCGTAGTTCAACCCGATCCGAACTCTGTGACAG  GGTTGAGACCTGGACCTGACCAAGGAGTAGCAGCAAATGGAGTTGGAGAAGCTGCCGGACCTCGAGGACAGTCGGAAAAT  GAGAACATAGACCAGGGTGGCAACCCTACACAGATTCCCAGACAACCTGAGCCTCAGCAAGCCGAAGCCGGTGCTACTCC  TGGAACTCTGAATTCATTCAGTTCATTGTTATTATGGATCTTGGGAGGTGCTTCATCCGAAGGCCGCAATTCGTTCCTTT  CCATGTTTAGAGATGTAAGAGAGCAAGGGCAGGTCTATACCGATCCCCCAAACGAAAATCGTGGAACGCAGAACGTGCAG  TAGCCGGGTTGCCTTTACGAAAGGGATGAAGAGGGTAGAGTTTAGCATTTAGGTGTATGTTGTATAGCTAATGTTCTAAT  CATATGCAAAACCTCTGAACAGTTTGGATATAACCACCCTAGGAACCTGTTATATAACTTGACATTGATCACAAATTAAG  GCTTAGCCCTTTCTTTTCTTTG |
| F01_transcript_4187. cDNA (3085 bp) |
| GGAAAGCGAAAATTCTTCGTCCTCGTCTTTCTCGTCATCAGAGCTTGGTTTATACGTCCGTCCGTTGCCCAAATCTACAA  ACAAATAAAGGCCAAGTGACGGTGGCGCCGCCTCCATGTCCTTACTCCACCGCAATCCTTTGCCGTCGTAATTTCTTTTT  GGTATTACGTGTTAAGAATTCCATTTTTTTTAAATGTTTTAAAAAATAATTTATCCGCTTGATTTGTTATTATTTTTAAA  TCTTTGGAGCTATTTTTTTGTTCGAACCGAATACCCGGACTGGAATTCTGCCATGGAAGGACCCGAAACGAGCTTGATTG  AAGTTAGAGAAGAGCTCATGGTTACACCAGGTCGCGAAACCCTAAACCCTTGTTCAAAAACAGCTCATTTCCTCAGACCC  ATTTTCAGTTCCCTCGAAACCCCACTTCCGAAGCTTCCTTCGCAACGTGTTTCGTTTCGGGAACAATCCTGTTTCGAACC  CAAAGATCTGCCGTTGAGTATCGGCTTCCATGGGTGGAGATGCAGGGCAAGTAATTGGTCCATCTGGGTTGACAAAATGA  GGGTTTTGCATGAATCGACTTGGAAGAAAGCTGGAATCTTCGAACCCATCTTGAATTCGACTTACCAGATCAAGCGAAAC  ACTGATTTAGTTCTTGGGTTAGCTGAGAAATGGTGTTGTGACACCAAAAGTTTCATTTTTTCTTGGGGCGAAGCCAGTGT  TACGCTCGAAGATGTCATGATTCTTGGGGGGTTTTCCGTTTTGGGTCCCTCTGTTTTCGCCCCGCTCGATTCCGATGACT  CTAGGGAAGTCGAAGCGAGTTTAAAGAGTGCGAGGATGGAAATCGTGAGAAGCAAAGCTAAAAAGGCCTGTCCTCGTTTG  TGGATGCAGAGATTCATGGACAATGGCTGCGAATTCGAGCACGAAGCTTTCCTCGCCTTTTGGCTTTCGAGGTATGTTTT  CACCAATGCGTATGAAACGATTAGGGAGCATGTTTTCGGCATTGCAGTTCATTTAGCTAGAGGGACAAGGCTTGCTTTAG  CACCCGCTGTGCTTGCTAGTATTTATAGAGACTTGTGTTTGTTGAAAGATGCTATGAATGCTTCCATTAAAATGGGGAAA  GATGAGGTGTTTAAGCTTACCCTTTGGTCGCCGTTTCAATTAGTTCAGGCTTGGGCTTGGGAGAGATTTTCTGAGCTGAG  ACCTCAACCGAATCCAATAGCAAAAGGTGAGCCAAGGCTGGTTCAATGGCATGATGTGAGTTGCAAGGTTGAGAATGTGA  GGTTAGCTCTAGAGTCGGCTAGTGGAAGCTTCGAATGGCGTCCTTATACTATGCAGATCAATAACTGGAAACAACCTAAG  TTTTATAGGGAGAATGAAGTTTGTATATGGACCACTGCCCGACTCGATAAAGAACTAGAGTCCTATGTCCGATGCTTGAA  GGTTAGTGAGCTAGTTGGAGTTGATTGCATAGAACAGTACCTTCCGCATCGAGTTGCAAGGCAATTTGGAATGGATCAGG  ATATTCCAGGTTGTGTTCCTCAATCGAAAAATCAGACTCATGAGATTGCCTGGCTCAATTACTGTGAATCGCCTACCGGT  GTAAAATTATACATTCCATCTCGGCTTTATAAAGCTGGTGTTACAGCGCGGTACCTGAAGTGGTGGAAGGATTCGGTGTT  GGAGAGCAAAGGTACGGCTCAGGGTTTGAAGAAATCGGCAAAGAATTCTAAAGGAAAGAAGCAAGGGAAAAATTCTTCAG  GTTGCTCTGGCTTTCATCAGAAAATAGAGAGTTTCCATGGAAAGACTGAAGCTATCGATCCATCGGTTTCTCCTAATTGC  ACCGTGAAGAGCTTAAAGAAACAAGGAGACACTGTCAAGAAAACTAAGAAATCAAAGAGTGTATCCAAGATTTTGGAAGA  AAAGGAAATGTGCAACAACGAATCTCCTTCTGCCTCTTGTAGAAAGAACTCTAAGAAATCAGCGAAGAATCCGAAAGGAA  ACAAAAAGGGCCGGGAAGAATCTTCTTCTCCCGAGGTTCCGTTGGGAAGCTCTAAAGAATCAGCTCAAACTTTGAAAAGA  AAGAAGAGAGACGATGGTGATTCTGCTTCCACTAGTTGGAGTAAAAAACACATGAAAAAGTCTGCAGAGAAATCTAAAGG  AATGAAGAAAGATACTTCTGTGCCTGCCTCTTCTAGATCCGCTTCCGGACACTCCAAAAAACGACCATTGGTTATGAAAG  ACAAGGAGGAAGCTAGCAGAGATGTCGCCTCTCATGTTTTACCTTCAGTAAGTGCAAAAAAATCACCATGCACTGTAGAA  AGAAAGAACGGTCGCAGTTCCTTGGACCTTATCAGTCTGGATTGTATCTTTAATGGAAAGGAAGCAGAGGATTCTACTGA  AGATTATAACCCAACAATTGCAGAAATGATGAGATCCTGCAAGAAGCGTCGCAATATCAGAACTAAAGACTCTGACGATG  ATGGAATCCCATCAGGCCATTCCCAAGGTCTTTCGTCGACAATCGCAGACGATGAGGTTGTTAAATACTTGGACCCATTA  GTAATGTTGGCTGAAAAGGTTATCGAGGTCGAGGACGAATCCGTGCTAAGAGTAAGGGAAACTTTCGAAAGTCCCTACAA  GGACCAGAGAGAAGTAAAGATGGTGCAGGAGGAAGCTGTGATGGGAGAATCAGGAAGAACAGCGGAACGTGAAGAGGGGG  ACAACCCCGAACAACCAGTTCACGAAATGCTAAGCATCAACGGAGTAGAAGGAGAGTGCAGTTGCTATGCGGTCGACATA  CCGGGATTAACACTCGAAACTCGGATTAGCAGGCTTGAGAAACTGGTTGAAGAGCTAAAAGCAATGAGATCTGCCTGCAA  GTAGTCTCTAAGCCATTAGCATTTGTGCTTGACTCATTATGGTTTTTTTTTCCCTTCTTTTTTAATTTTCTCTGGTTGGT  TTGATTCAGTGGAAAGAATACCGTAAACTTGCTTGAGCCGAACATTGCATGTAACGAAAAATATGTTGTTGATTATTATA  AAGGGTAAACTATTCAGTTGGTCACCCAACTATCAAACTTTTTTT |
