# Supplementary material for: Integrated Analyses of Transcriptome and Chlorophyll Fluorescence Characteristics Reveal the Mechanism Underlying Saline–Alkali Stress Tolerance in Kosteletzkya pentacarpos
Source: Front Plant Sci. 2022 May 6;13:865572. doi: 10.3389/fpls.2022.865572 (PMC9122486; doi:10.3389/fpls.2022.865572)
Supplement: Supplementary file 5 [file Table_4.DOCX]

**Supplementary Table 4.** The Primer sequence of Real-time PCR for key genes of *K. virginica* seedlings under salt and alkali stress

| Primer name | Forward primer (5′—3′) | Reverse primer(5′—3′) |
| --- | --- | --- |
| F01_transcript_53932 | TGTTCTTGGTGCCGCTATGT | GAAGTTCCTGAGCACGACCA |
| F01_transcript_13312 | GCCGGGGCAATCAAGTTTTT | ATCAGAACCACCTTCAGCGG |
| F01_transcript_3631 | GCTGCGTTCATGTGGTGTTT | TCAGATCCCGGACAAGGCTA |
| F01_transcript_7879 | CAATGTAGCCGCGATCCAGA | ACCGCTCCACCTTTCTGTTT |
| F01_transcript_59507 | ATCCTTTGGCCGGAGATTGG | CTCCACACGAAGCCCTCATT |
| F01_transcript_25894 | GGTTGAGGGTGGCAGCTAAT | ATGAACACGGCCGAGGATTT |
| F01_transcript_95488 | GGAACTCCAACTCCGAAGGG | AGGCATCAACCGCAGACTAC |
| F01_transcript_9571 | TCGTCGCTTTCGACTGTTCA | CGTCTCCGTTCAACTTCCGA |
| F01_transcript_4187 | GCTTCCTTCGCAACGTGTTT | TTCGAGCGTAACACTGGCTT |
| β-actin | TTATGTTGCCCTGGACT | CCGCTTCCATCCCTA |
